# Construction of programmed time-released multifunctional hydrogel with antibacterial and anti-inflammatory properties for impaired wound healing

**DOI:** 10.1186/s12951-024-02390-y

**Published:** 2024-03-23

**Authors:** Yuan Peng, Yicheng Guo, Xin Ge, Yali Gong, Yuhan Wang, Zelin Ou, Gaoxing Luo, Rixing Zhan, Yixin Zhang

**Affiliations:** 1 Department of Plastic and Reconstructive Surgery, Shanghai Ninth People’s Hospital, Shanghai JiaoTong University School of Medicine, 639 Zhi Zao Ju Road, Shanghai, 200011 China; 2 Institute of Burn Research, State Key Laboratory of Trauma and Chemical Poisoning, Southwest Hospital, The Third Military Medical University (Army Medical University), Chongqing, 400038 China; 3 Department of Gastroenterology, The Second Affiliated Hospital of Chongqing Medical University, Chongqing, 400010 China

**Keywords:** Nanozymes, Time-released hydrogel, Antibacterial, Anti-inflammation, Wound healing

## Abstract

The successful reprogramming of impaired wound healing presents ongoing challenges due to the impaired tissue microenvironment caused by severe bacterial infection, excessive oxidative stress, as well as the inappropriate dosage timing during different stages of the healing process. Herein, a dual-layer hydrogel with sodium alginate (SA)-loaded zinc oxide (ZnO) nanoparticles and poly(N-isopropylacrylamide) (PNIPAM)-loaded Cu_5.4_O ultrasmall nanozymes (named programmed time-released multifunctional hydrogel, PTMH) was designed to dynamically regulate the wound inflammatory microenvironment based on different phases of wound repairing. PTMH combated bacteria at the early phase of infection by generating reactive oxygen species through ZnO under visible-light irradiation with gradual degradation of the lower layer. Subsequently, when the upper layer was in direct contact with the wound tissue, Cu_5.4_O ultrasmall nanozymes were released to scavenge excessive reactive oxygen species. This neutralized a range of inflammatory factors and facilitated the transition from the inflammatory phase to the proliferative phase. Furthermore, the utilization of Cu_5.4_O ultrasmall nanozymes enhanced angiogenesis, thereby facilitating the delivery of oxygen and nutrients to the impaired tissue. Our experimental findings indicate that PTMHs promote the healing process of diabetic wounds with bacterial infection in mice, exhibiting notable antibacterial and anti-inflammatory properties over a specific period of time.

**Supplementary Information:**

The online version contains supplementary material available at 10.1186/s12951-024-02390-y.

## Introduction

The classical process of wound healing encompasses four distinct stages: hemostasis, inflammation, proliferation, and remodeling, which are tightly interrelated and can significantly affect one another [1]. These stages are also characterized by time-sequence and continuity [2]. The regulation of the immune microenvironment plays a crucial role in facilitating the sequential advancement of these stages [3]. Nevertheless, impairment of wound healing due to dysfunction in immunoregulation can result in wounds that often remain in the inflammatory phase, especially the diabetic wound [4]. The impaired healing of diabetic wounds can also be attributed to the severe inflammation induced by bacterial infection [5], leading to the impairment of angiogenesis as well as excessive production of pro-inflammatory cytokines, such as interleukin(IL)-6, IL-1β and tumor necrosis factor-α (TNF-α) [6]. Moreover, uncontrolled inflammatory response can lead the infiltration of pro-inflammatory cell and the overproduction of reactive oxygen species (ROS) which damaged to normal cells and tissue [7, 8]. Hence, disruption of the immune microenvironment hinders the development of granulation tissue, neovascularization, and re-epithelialization in the healing process. Notably, the lack of an adequate inflammatory response during the initial stages is directly linked to uncontrolled bacterial infection and a weakened early immune response [9], resulting in impaired healing of cutaneous wounds. Consequently, there is a critical need for targeted manipulation of the wound microenvironment during the different phases of repair, particularly in the case of impaired wound healing, in clinical settings.

In recent years, extensive research has been conducted to explore various methodologies aimed at managing impaired wound healing by manipulating the inflammatory microenvironment at the site of the wound, including the utilization of nanoparticles [10], application of functional hydrogels [11, 12], and implementation of treatment with anti-inflammatory drugs [13]. Numerous studies [14–16], including our studies [8, 17], have constructed various nanomaterials with ROS scavenging effect for inflammation-related disease treatment. These approaches have been associated with the promotion of wound healing. Nevertheless, the failure to consider the immune-related attributes associated with each stage of wound healing impedes the efficacy of active constituents, thereby constraining the therapeutic effectiveness. It is demonstrated that, in the early stage of inflammation, proinflammatory macrophages (M1 types) produce moderate levels of ROS and pro-inflammatory factors, such as IL-6 and TNF-α, to exert bactericidal effects [18]. In contrast, complete inhibition of M1 macrophages at the early stage may aggravate bacterial infection and delay wound healing. Despite the existence of numerous antibacterial agents, the healing of diabetic wounds cannot be solely achieved through their use, owing to persistent and excessive inflammation [19]. Therefore, a dynamical programmed hydrogel with microenvironment moderation corresponding to distinct healing stages need to be explored.

**Fig. 1:**
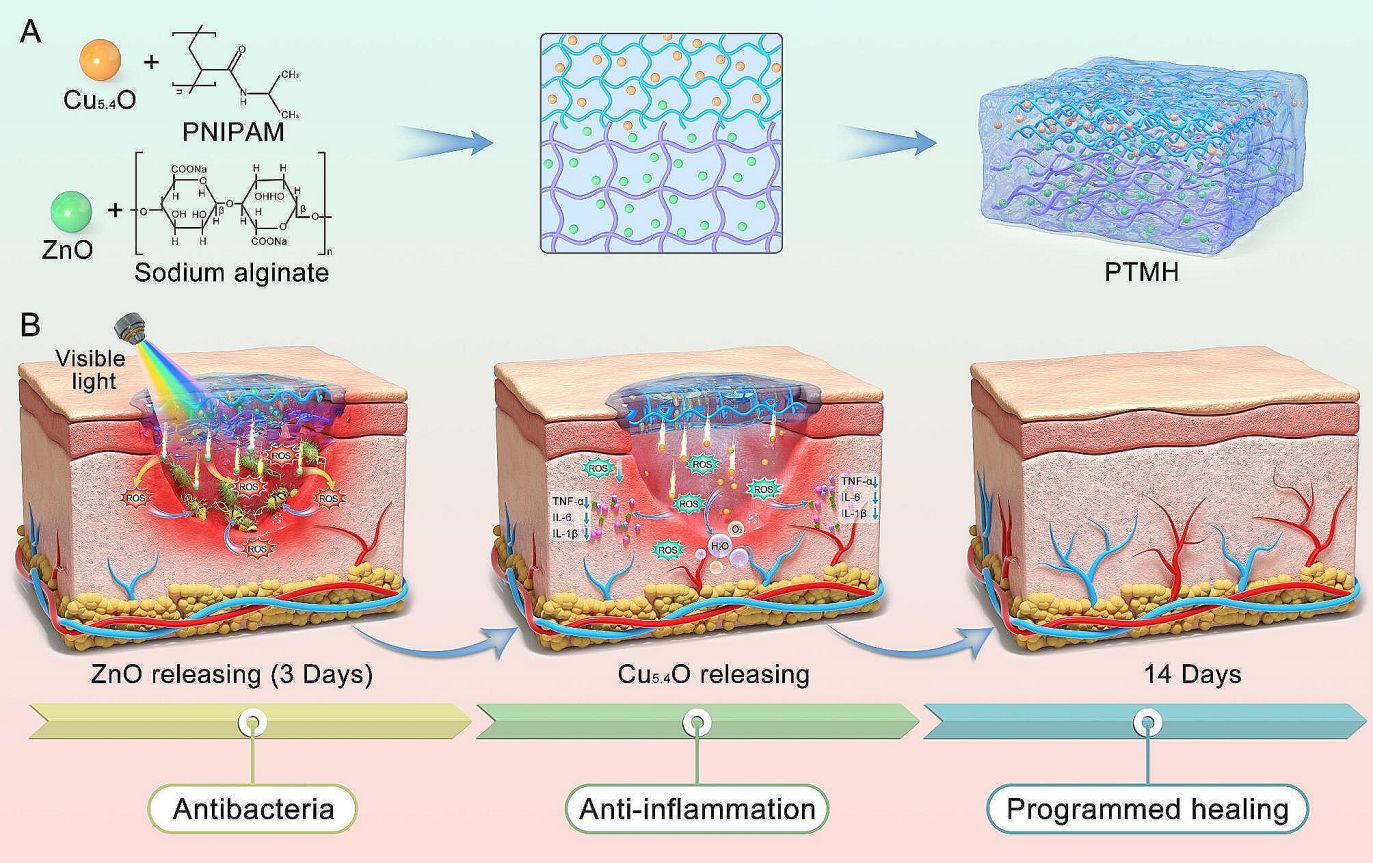
Schematic of the design, structure, and therapeutic process of PTMH for impaired wound healing. (A) A dual-layer hydrogel with sodium alginate (SA)-loaded zinc oxide (ZnO) nanoparticles and poly(N-isopropylacrylamide) (PNIPAM)-loaded Cu_5.4_O ultrasmall nanozymes was designed. (B) The programmed regulation process in the mouse model of diabetic wounds with *P. aeruginosa* infection, including anti-infection with ZnO via generation of reactive oxygen species (ROS) and anti-inflammation, as well as angiogenesis promotion by Cu_5.4_O ultrasmall nanozymes. PTMH, programmed time-released multifunctional hydrogel

In the present study, programmed time-released multifunctional hydrogels (PTMH) were constructed to inhibit the growth of bacteria at the early stages and alleviate inflammation at the wound site in time-sequence according to the characteristics of the distinct stages of wound healing (Fig. 1A). To meet the requirements of the classical wound healing process, PTMH consisted of two main parts, namely sodium alginate (SA) zinc oxide (ZnO@SA) hydrogel and poly(N-isopropylacrylamide) (PNIPAM)-loaded Cu_5.4_O (Cu_5.4_O@PNIPAM) hydrogel. Following the occurrence of tissue injury, ZnO@SA could provide hemostasis [20] and release ZnO nanoparticles that possess strong antibacterial activity under visible light (VL) irradiation by producing moderate oxygen free radicals. Moreover, ROS may activate M1 macrophages and increase the clearance ability [18], which are crucial for bacterial elimination, corpse clearance, and sequenced repair process activation within the first three days postinjury. Three days postinjury, the structure of the lower part of the PTMH (ZnO@SA) collapsed, and the Cu_5.4_O@PNIPAM hydrogel came in direct contact with the wound area. The Cu_5.4_O@PNIPAM hydrogel could sustainably release Cu_5.4_O nanozymes to scavenge excessive ROS at the wound site and prevent further signaling for the activation of inflammation during the stages of repair (Fig. 1B). PNIPAM could also provide a contraction force to promote wound healing [21]. Our study aimed at the different characteristics of the distinct stages of wound healing in time-sequence. Proregenerative potential of PTMH hydrogels were evaluated in mouse models of impaired diabetic wounds with bacterial infection.

## Methods

### Materials and instruments

Dihydroethidium (DHE) was provided by Sigma‒Aldrich Co., Ltd. (Saint Louis, USA). Dulbecco’s modified Eagle’s medium (DMEM) and fetal bovine serum were purchased from Hyclone (Logan, USA). Murine tumor necrosis factor-alpha (TNF-α), interleukin 6 (IL-6), IL-1β, IL-10, vascular endothelial growth factor (VEGF), superoxide dismutase (SOD) and myeloperoxidase (MPO) enzyme-linked immunosorbent assay (ELISA) kits were provided by Sangon Biotech Co., Ltd. (Shanghai, China). Rabbit anti-CD31 and rabbit anti-proliferation cell nuclear antigen (PCNA) antibodies were purchased from Cell Signaling Technology Co., Ltd. (USA). Goat anti-rabbit-Alexa Fluor-555 antibody was obtained from eBioscience Co., Ltd. (USA). Rat anti-mouse LY6G-FITC, CD86-Brilliant Violet 421™ and CD11b-Brilliant Violet 650™ antibodies were obtained from BioLegend Co., Ltd. (USA). Immunol staining primary antibody dilution buffer and 2′,7′-dichlorofluorescin (DCFH) diacetate probe were purchased from Beyotime Co., Ltd. (China). Liberase™ TL Research Grade was purchased from Roche Co., Ltd. (Switzerland).

Male BALB/c mice (age: 8–10 weeks; weight: 20–25 g; *n* = 60) were obtained from the Experimental Animal Department of the Army Medical University (Chongqing, China) and were housed under pathogen-free conditions in experimental animal cages for 1 week before colitis model establishment under the following conditions: 12:12-h light and dark cycle; constant temperature of 22 °C ± 1 °C; relative humidity of 45–55%; and free access to autoclaved chow and water. All animal operations were performed under anesthesia with 1% sodium pentobarbital solution (50 mg kg^− 1^, intraperitoneally) to minimize suffering. Animal experiments were approved by the Laboratory Animal Welfare and Ethics Committee of the Army Medical University (approval number: AMUWEC20226325).

The morphology of the nanoparticles was investigated by transmission electron microscopy (TEM, HT7800, Japan). The hydrodynamic size and surface zeta potential of the nanoparticles were measured using a dynamic light scattering (DLS) instrument (Zetasizer Nano ZS90, UK). Hydrogels were irradiated using a Light source (CEL-PE300-3 A; Beijing China Education Au-light Co., Ltd.) The cells and tissues samples were photographed using a laser confocal microscope (Zeiss LSM780, Germany).

### Synthesis of PTMH

#### Cu_5.4_O@ PNIPAM

Cu_5.4_O nanoparticles were synthesized based on our previous work [8]. For the thermoresponsive layer, 1.06 M N-isopropylacrylamide (NIPAM), solution in phosphate-buffered saline (PBS) was mixed with 0.28 mM N,N′-methylenebisacrylamide, 6.5 mM initiator ammonium persulfate, and 3.7 mM accelerator tetramethylethylenediamine using a syringe. The hydrogels were gelled overnight inside a closed glass mold at 20 °C. To prepare Cu_5.4_O@PNIPAM hydrogels, Cu_5.4_O was loaded into gels by soaking method. Briefly, the prepared gels were immersed in PBS solutions containing Cu_5.4_O at the concentrations of 1–60 µg mL^− 1^ for 2 h. Non-loaded Cu_5.4_O nanoparticles was removed by washing with PBS for five times. For ZnO synthesis, 12 mM Zn(Ac)2·2H_2_O was dissolved in 80 mL ethanol and stirred for 2 h at 80 °C. Next, 35 mM KOH was dissolved in ethanol (2 mL); and a certain amount of KOH solution was added dropwise into the above solution. Subsequently, (3-aminopropyl) triethoxysilane (250 µL) was added, and the solution was stirred overnight at room temperature. Finally, the ZnO nanoparticles were collected by centrifugation, washed with absolute ethanol thrice, and dried in an oven blast at 40 °C for 12 h. The adhesive surface was prepared with 15% (w/w) sodium alginate, 4% (w/w) gelatin methacryloyl, 0.2% (w/w) α-ketoglutaric acid, and ZnO nanoparticles (100–1000 µg mL^− 1^) in deionized water, cured in an ultraviolet light chamber (365 nm, 6 W power) for 30 min, and subsequently soaked in calcium chloride solution (2 M) for 6 h. The mixture of SA, 1-ethyl-3-(3-dimethylaminopropyl) carbodiimide, and N-hydroxysuccinimide was applied to the surface of the hydrogel matrix prior to application.

#### Mechanical tests

The stress-strain curve was obtained using the Instron machine; the strength test was conducted with a strain rate of 100 mm min^− 1^.

#### Rheological analysis

Rheological measurements of the adhesive were performed with a TA DHR-2 rheometer. The oscillation strain dependence of the storage modulus G′ and loss modulus G″ was detected at a fixed strain of 0.5%, which ensured sufficient sensitivity within the region of linear viscoelasticity. All tests were performed at 20–45 °C.

#### ROS production under visible light

The peroxidase-mimicking activity of ZnO was determined using the oxidation of 3,3′,5,5′-tetramethylbenzidine. The experiments were performed in PBS (pH 4.0, 25 mM), and measurements were conducted using ultraviolet-visible absorption spectroscopy at 652 nm. VL was used to examine the light activation ability. Additionally, absorbance at 652 nm was measured on following 3 days.

#### ROS scavenging test by Cu_5.4_O@PNIPAM

To determine the ability of scavenging ROS by Cu_5.4_O@PNIPAM, gradient concentration of Cu_5.4_O@PNIPAM (1 − 32 ng µL^− 1^) were introduced. For H_2_O_2_, ·OH and O_2_·^−^ assay, samples were co-incubated with work solution, then the absorbance at 550 nm, 652 nm and 550 nm was measured, respectively. For DPPH and ABTS free radicals scavenging test, 500 µM DPPH and 2.45 mM ABTS were co-incubated with samples and the absorbance at 517 and 734 nm was measured, respectively.

#### Clotting time assay

A sample of citrated blood was obtained and transferred into an Eppendorf tube. Subsequently, a solution of 0.1 M calcium chloride was introduced into the tube and thoroughly mixed with the blood for a duration of 10 s. Following this, a volume of 50 µL from the mixture was carefully dispensed into individual wells of a 96-well plate. At specific time intervals, each well underwent a thorough rinsing with a saline solution, and the liquid was promptly removed. Time required for the formation of a uniform clot in each well was meticulously recorded. Additionally, at certain predetermined intervals, a saline solution with a concentration of 9 g/L was employed to cease the clotting process by rinsing each well. The liquid was promptly aspirated, and the wells underwent multiple washes until the solution achieved clarity, signifying the elimination of all soluble blood components. In preparation for scanning electron microscopy (SEM), the samples were fixed in a 2% glutaraldehyde solution for a duration of 12 h firstly. Then, the samples were in immersed in 50%, 70%, 80%, and 95% ethanol for 5–7 min at a time, and 100% ethanol dehydration twice. Next, the samples were dehydrated by 50%, 70%, 80%, and 95% tert-butanol for 5–7 min, and 100% tert-butanol dehydration twice. Finally, when tert-butanol volatilized in 4 °C refrigerator, the samples were coated with a layer of gold via sputter-coating, and subsequently analyzed utilizing SEM.

#### Cell culture

NIH-3T3 mouse embryonic fibroblasts (3T3) and human umbilical vein endothelial cells (HUVECs), obtained from the American Type Culture Collection (ATCC), were cultured in DMEM supplemented with 10% fetal bovine serum (FBS), 100 µg mL^− 1^ streptomycin, and 100 U mL^− 1^ penicillin in a cell incubator (5% CO_2_, 37 °C).

#### The antibacterial activity of PTMH in vitro

To evaluate the antibacterial effect of PTMH, a gram-negative bacteria *P. aeruginosa* (ATCC 27,853) and a gram-positive bacteria methicillin-resistant Staphylococcus aureus (MRSA) (ATCC 43,300) were chosen. For bacterial suspension test, bacterial colony was cultured in Luria–Bertani medium at 37 °C for 12 h. Subsequently, the suspension was diluted with LB medium to achieve the initial concentration (OD_600_ = 0.07), and bacterial suspension was transferred to cell culture plate (500 µL per well). Then, bacteria were co-incubated with a hydrogel (ZnO@SA, Cu_5.4_O@PNIPAM, and PTMH, respectively) with or without VL treatment (*n* = 5) at 37 °C for 24 h with shaking and the OD_600_ was recorded using a microplate reader (Thermo Varioskan Flash, USA). Furthermore, the bacterial suspension underwent serial dilution using saline solution to achieve a 2,500-fold dilution for *P. aeruginosa* or a 10,000-fold dilution for MRSA. Subsequently, the diluted bacterial solution (25 µL) was evenly distributed on an agar plate and were incubated in an incubator for 18 h. The resulting bacterial colonies were captured in photographs and their quantity was enumerated. The control group for this experiment consisted of a bacterial solution that received no treatment.

To further determine the underlying mechanism of the antibacterial effect of PTMH, the cellular ROS levels were detected using the DCFH probe. Briefly, 3T3 cells were cultured in 24-well cell culture plates and coincubated with ZnO@SA and PTMH with or without VL treatment (*n* = 5). Cells treated with PBS were used as control. Next, cells from all groups were coincubated with a DCFH probe diluted to 1:1000 for 30 min at room temperature. Next, fluorescence imaging was photographed with a laser confocal microscope (Zeiss LSM780, Germany). The fluorescence intensity was analyzed using the ImageJ software. In addition, the distribution of DCFH positive 3T3 cells was determined with flow cytometry.

#### Evaluation of the antioxidative stress effect of PTMH in vitro

The 3T3 cells and HUVECs were cultured in 24-well cell culture plates before incubation with 250 µM H_2_O_2_ (*n* = 5). Thereafter, ZnO@SA, Cu_5.4_O@PNIPAM, and PTMH were added to the plates, followed by coincubation for 24 h.

#### Cell-protecting effect test

The cell-protecting effect of PTMH was determined using CCK-8 assay. Briefly, CCK-8 working solution (50 µL) and DMEM cell culture medium (450 µL) were added to each well of the culture plate. After incubation for 1 h, the absorbance of each group at 450 nm was recorded using a microplate reader (Thermo Varioskan Flash, USA).

#### In vivo hemostatic sealing of rat liver

For the analysis of hemostasis, the rats were administered 1% pentobarbital anesthesia. Pre-weighed filter paper was positioned beneath the liver. An injury of 6 mm diameter and 3 mm depth was made to the liver using a biopsy punch (Dynarex). Immediately, different treatments were used. The weight of the filter paper containing blood was assessed (*n* = 5).

#### Evaluation of wound healing using a model of infected diabetic wound

A mouse model of diabetes disease was firstly established. Briefly, after high-fat and high-glucose feeding for 10 days, male BALB/c mice (age: 8–10 weeks; weight: 20–25 g) were intraperitonially administered 10 mg kg^− 1^ streptozotocin (STZ, Sigma‒Aldrich, USA) diluted in sodium citrate buffer at a concentration of 10 mg mL^− 1^ continuously for 5 days. Then, the mice were fed a high-fat and high-glucose diet for an additional 10 days. Blood glucose levels were measured every 2 days using a glucometer. The mice with blood glucose levels in excess of 16.7 mmol L^− 1^ were considered diabetic. To establish model of the infected wound, diabetic mice were initially anesthetized with 1% pentobarbital sodium (60 mg kg^− 1^). Subsequently, two round wounds were created on the backs of the mice. Each wound was treated with a 20 µL solution of *Pseudomonas aeruginosa* and the hydrogels were fixed to the wound with 3 M Tegaderm™ wound dressing (3 M Health Care, USA). After one day of feeding, the mice were treated with ZnO@SA, Cu_5.4_O@PNIPAM, and PTMH (*n* = 5). Wounds treated with PBS served as control. The hydrogels were not replaced during the entire period of the assay. Images of the wounds were captured at 0, 3, 7, and 14 days postsurgery.

### Histological examination

#### Hematoxylin and eosin (H&E) staining

Tissue from the wound site was harvested (*n* = 5), fixed with 4% paraformaldehyde for 24 h, embedded in paraffin, and cut into sections (thickness: 7 μm). Following a 30-minute deparaffinization process in xylene, the sections underwent rehydration using a descending ethanol series (100%, 90%, 80%). Subsequently, the sections were immersed in hematoxylin for a duration of 5 min, followed by a 3-minute rinsing with PBS. Eosin immersion lasted for 2 min, followed by a 5-minute immersion in distilled water. Dehydration was then carried out using ethanol/water mixed solutions (80%, 90%, and 100%) for 5 min, followed by a 15-minute immersion in xylene. Finally, the sections were mounted on slides using neutral resin and coverslips. Image capture was performed using an Olympus SLIDEVIEW VS200 (Nikon, Tokyo, Japan).

#### Masson’s trichrome staining

The paraffin sections underwent deparaffinization and rehydration, and were subsequently stained using a Masson’s trichrome staining kit in accordance with the manufacturer’s instruction. Images were acquired using an Olympus SLIDEVIEW VS200 microscope (Nikon, Tokyo, Japan).

#### Immunofluorescence staining

The paraffin sections were deparaffinized and rehydrated as described above, and coincubated overnight with rabbit anti-mouse CD31 and PCNA at 4℃. Thereafter, the sections were incubated with goat anti-rabbit Alexa Fluor 594 (Abcam, Cambridge, MA, USA) secondary antibodies at room temperature for 1 h. Fluorescent images were photographed using a laser confocal microscopy (Zeiss LSM780, Germany).

#### ROS detection of wound tissue

Wound tissues were harvested at 3 and 14 days postsurgery (*n* = 5). To evaluate ROS levels, the tissues were embedded in optimal cutting temperature (O.C.T.) matrix for cryostat sectioning. Next, sections were coincubated with DHE (1:400 dilution) at room temperature for 30 min. Fluorescent images were photographed using a laser confocal microscopy (Zeiss LSM780, Germany).

#### Identification of M1 macrophages by FACS

At 3 and 14 days postsurgery, wound tissues from each group were harvested and digested using Liberase™ TL (Roche, Switzerland) for 2 h at 37℃ (*n* = 5). The cell pellets were collected after centrifugation at 1,500 rpm for 5 min. Next, the cells were coincubated with rat anti-mouse CD11b-Brilliant Violet 650™, LY6G-FITC, and CD86-Brilliant Violet 421™ (1:200) at 4℃ for 1 h. Thereafter, the percentage of M1 macrophages was determined with flow cytometry.

#### Quantitative detection of cytokines from wound tissue

Wound tissue from each group was harvested, homogenized at 4℃, followed by centrifugation at 12,000 rpm for 20 min at 4℃ (*n* = 5). Then, the supernatants were collected, and the concentrations of TNF-α, IL-6, IL-1β, IL-10, VEGF, SOD, and MPO were quantified by the corresponding ELISA kits.

### Transcriptome analysis and western blotting

Mouse cutaneous wound samples for transcriptome analysis were obtained from tissues on day 14 of treatment with 3 M Tegaderm™ wound dressing or PTMHs (*n* = 3). Total RNA was extracted from the tissues using the RNeasy Fibrous Tissue Mini Kit. Subsequently, the quality of RNA was tested using a 2100 Bioanalyzer (Agilent). The concentration of RNA was determined using a NanodropND-1000 spectrophotometer (Gene Company, USA). Pure RNA samples were severed as initial material to prepare libraries and for sequencing using Illumina HiSeq X10 (Illumina, USA).

The bioinformatic data were analyzed using the Majorbio Cloud Platform (Majorbio Biopharm Biotechnology, China). Sequencing quality was assessed using Fastx-Toolkit v.0.0.14. All reads were mapped to the reference genome of Mus_musculus v.GRCm38.p6 (http://asia.ensembl.org/Mus_musculus/Info/Index). Gene abundances were quantified through RSEM v.1.3.1 (http://deweylab.biostat.wisc.edu/rsem). Analysis of differential expression was achieved using DESeq2 v.1.24.0 (http://bioconductor.org/packages/stats/bioc/DESeq2/) (cutoffs: fold change ≥ 2 and *p*-value < 0.05). Heatmaps were generated, and hierarchical clustering was conducted using Perseus v.1.6.1.1 (http://www.coxdocs.org/doku.php?d=perseus:start). Kyoto Encyclopedia of Genes and Genomes (KEGG) functional enrichment analysis was carried out using KOBAS v.2.1.1 (http://kobas.cbi.pku.edu.cn/download.php).

### Biocompatibility evaluation

Initially, the biocompatibility of PTMH was detected using CCK-8 kits in vitro. HUVECs and 3T3 cells were cultured in cell plates (density: 5 × 10^4^ cells per well) one day prior to the experiment. Next, ZnO@SA, Cu_5.4_O@PNIPAM, and PTMH were introduced into the upper chamber of the Transwell system (*n* = 5). After 24 h of coincubation, the rest of medium was discarded, and fresh culture medium (450 µL) and CCK-8 solution (50 µL) were added. After incubation for 1 h, the absorbance of each group at 450 nm was recorded using a microplate reader (Thermo Varioskan Flash, USA).

To further assess the biocompatibility of PTMHs in vivo, PTMH hydrogels were subcutaneously implanted into male BALB/c mice (age: 6–8 weeks; weight: 20 − 25 g; *n* = 5). Mice treated with PBS served as control. On 30 days post-treatment, blood samples were obtained and underwent complete blood tests and serum biochemistry analysis, including the assessment of blood urea nitrogen(BUN), creatinine (CRE), alanine aminotransferase (ALT), and aspartate aminotransferase (AST). Finally, mice from the control and PTMH groups were sacrificed on 3 and 30 days post-treatment, respectively, and major organs (e.g., heart, liver, spleen, lungs, and kidneys) were harvested to produce tissues for H&E staining.

### Statistical analysis

Data are presented as the means ± standard deviations of independent measurements (*n* = 3 or 5). One-way analysis of variance (ANOVA) or two-tailed Student’s *t*-test was used for statistical analysis in GraphPad Prism 5 software (GraphPad Software Inc., La Jolla, CA, USA). The *p*-values < 0.05 indicate statistically significant differences.

## Results and discussion

### Characterization of hydrogels

**Fig. 2:**
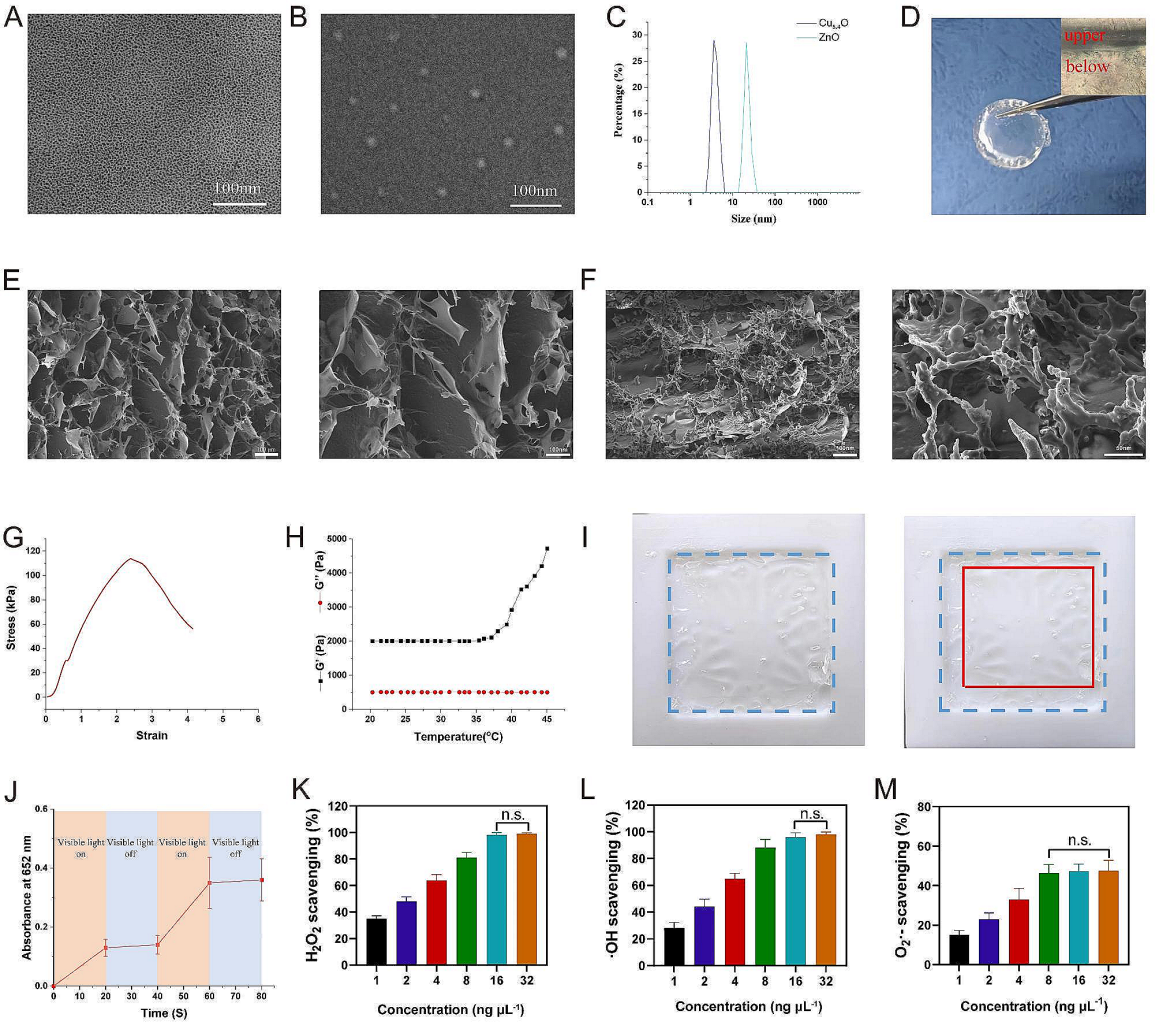
Preparation and characterization of PTMHs. (A) TEM images of Cu_5.4_O and (B) ZnO. (Scale bar: 100 nm). (C) The particle size distribution of Cu_5.4_O and ZnO nanoparticles. (D) Optical image of PTMH. (E) SEM images of PTMH. (Scale bar, left: 100 µm; right: 100 nm) (F) SEM images of ZnO@SA layer view after 3 days of incubation at 35°C. (Scale bar, left: 100 nm; right: 50 nm). (G) The stress‒strain curve of PNIPAM. (H) The elastic modulus (G’) and loss modulus (G’’) of PNIPAM. (I) Images of PNIPAM following incubation at 35 °C. (J) The test of ROS production of ZnO. (K) H_2_O_2_, (L) ·OH, and (M) O_2_·^−^ scavenging capacity of different concentrations of Cu_5.4_O@PNIPAM. Data in K, L, and M represent the mean ± standard deviation (*n* = 5). n.s., no significance; one-way ANOVA. TEM, transmission electron microscopy; SA, sodium alginate; PNIPAM, poly(N-isopropylacrylamide); PTMH, programmed time-released multifunctional hydrogel; ANOVA, analysis of variance

As shown in Fig. 1, the hydrogel was composed of two layers. The upper layer was Cu_5.4_O-loaded thermoresponsive PNIPAM, and the lower layer was a ZnO@SA hydrogel. In our design, upon application of the as-prepared hydrogel, the ZnO@SA layer provides hemostasis and antimicrobial function, and ZnO nanoparticles could produce ROS to improve the antibacterial ability via photocatalytic activity under VL [22]. Subsequently, the upper layer actively contracted wounds by shrinking at approximately 32°C, and the Cu_5.4_O nanoparticles scavenged ROS at the scheduled time. Figure 2A–B show transmission electron microscopy images of Cu_5.4_O and ZnO, respectively. Both Cu_5.4_O and ZnO nanoparticles were uniform, with values of ∼ 5 and 20 nm, respectively. The particle size distribution of Cu_5.4_O and ZnO nanoparticles was measured using DLS. This analysis showed that the particle sizes were approximately 7 and 25 nm, respectively (Fig. 2C). The optical image of this hydrogel is shown in Fig. 2D, and the inset is the optical microscope image. There was a clear boundary between the upper and lower layers, which demonstrated that the two-layer hydrogel patch was well constructed. Figure 2E shows SEM images of the hydrogel, revealing a good pore structure. Figure 2F shows the hydrogel SEM image after degradation of the lower layer three days postincubation at 37°C; notably, the pore structure of the lower layer no longer existed at that point. This result is consistent with our expectation because we require the upper layer to exert its function after three days. The stress‒strain curve shows that the PTMH possess good mechanical strength (Fig. 2G). The skin of the adult human loss the ability to contract wounds, which has been proved a key role in embryonic wound healing. Therefore, the skin temperature response mechanically active adhesive dressings were designed to actively contract wounds to promoting wound healing. In the temperature-dependent rheological test (Fig. 2H), the elastic modulus (G’) and loss modulus (G’’) changed by approximately 32 °C, which is evidence of the phase change of the PNIPAM-based hydrogel. Figure 2I demonstrates that the hydrogel could shrink at 35 °C, as expected, to actively contract wounds. We conducted an on-off experiment to examine the ROS production ability via photocatalytic activity under VL (Fig. 2J). Following exposure to VL, ZnO exhibited peroxidase-like activity (ultraviolet-visible spectrophotometry spectrophotometer at 652 nm wavelength) [22]. This suggests that the as-prepared ZnO possesses VL activation ability. However, the ability for ROS production gradually decreased with time, and this ability was lost at approximately three days (Fig. S1).

H_2_O_2_, being a prevalent endogenous ROS variant, was initially employed to examine the ROS elimination capability of Cu_5.4_O@PNIPAM. The results indicated an obvious reduction of H_2_O_2_ following incubation with escalating concentrations of Cu_5.4_O@PNIPAM, ultimately achieving a rate of 98.3% at 16 ng µL^− 1^ (Fig. 2K). ·OH radicals are among the most potent and biologically relevant free radicals, exerting a significant influence on inflammation disorders. The scavenging ability of Cu_5.4_O@PNIPAM toward ·OH radicals was gradually enhanced with the escalating concentration of Cu_5.4_O@PNIPAM, ultimately achieving a remarkable scavenging rate of 96% at 16 ng µL^− 1^ (Fig. 2L). Similarly, the O_2_·^−^ scavenging test showed that Cu_5.4_O@PNIPAM could scavenge 47.3% of O_2_·^−^ at a concentration of 16 ng µL^− 1^ (Fig. 2M). Moreover, to investigate the free radical scavenging ability, 2,2′-azino-bis(3-ethylbenzthiazoline-6-sulfonate) (ABTS) radicals were chosen. The results revealed that the scavenging rate could reach 96% following treatment with Cu_5.4_O@PNIPAM (Fig. S2). Additionally, Cu_5.4_O@PNIPAM also showed an efficient scavenging ability towards 2,2-diphenyl-1-(2,4,6-trinitrophenyl) (DPPH) radicals (Fig. S3). To further determine the cytoprotective ability of PTMH, 3T3 cells and HUVECs were pretreated with ZnO@SA, Cu_5.4_O@PNIPAM, and PTMH. Next, the cells were incubated with 250 µM H_2_O_2_. Following Cu_5.4_O@PNIPAM and PTMH treatment, cell viability remained the higher level compared with that of the ZnO@SA and H_2_O_2_ groups, which was attributed to the ROS scavenging effect of Cu_5.4_O (Fig. S4). The results demonstrated that Cu_5.4_O@PNIPAM could efficiently scavenge ROS and free radicals and protect cells from excessive oxidative stress damage, which is consistent with those of our previous research.

Furthermore, according to the in vitro cytotoxicity results (Fig. S5), we determined relatively safe concentrations of ZnO@SA, Cu_5.4_O@PNIPAM, and PTMH for subsequent biological experiments. There was no significant difference in cell viability between the hydrogel treatment group and control group (*p >* 0.05). These data indicated that the hydrogel is safe for cells.

### Antibacterial activity of PTMH

**Fig. 3:**
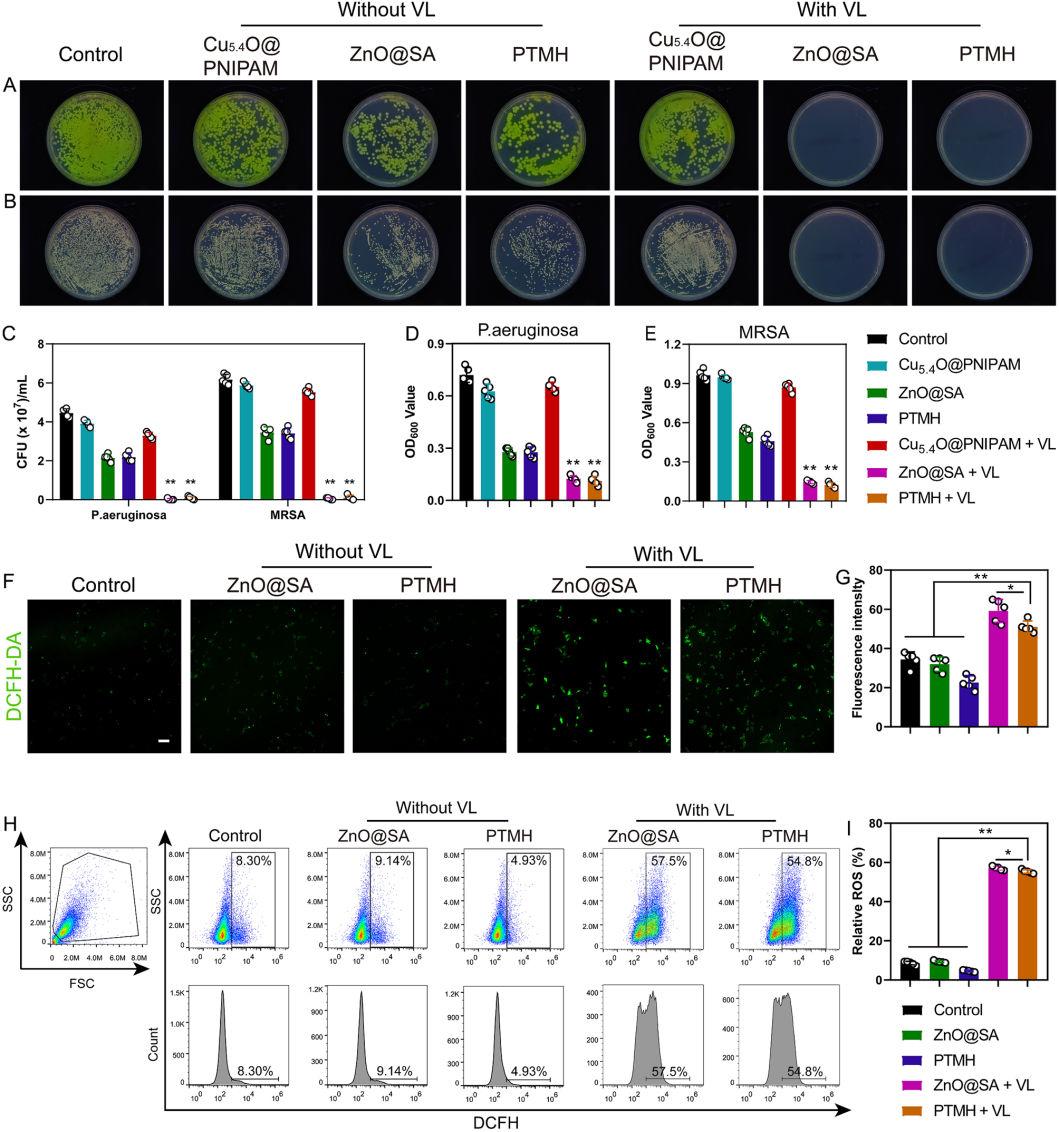
Evaluation of the antibacterial activity of PTMH in vitro. (A) Images of bacterial colonies of *P. aeruginosa* and (B) MRSA following treatment with Cu_5.4_O@PNIPAM, ZnO@SA, and PTMH with or without VL irradiation. (C) The number of bacterial colonies formed by *P. aeruginosa* and MRSA under different treatments. (D) OD_600_ values of *P. aeruginosa* and (E) MRSA under different treatments. (F) Representative confocal digital images and (G) fluorescence intensity for DCFH-DA (green) staining of 3T3 cells under different treatments. (Scale bar: 50 μm) (H) ROS levels and (I) statistical analysis in 3T3 cells under different treatments. Data in C–E, G, and I represent the mean ± standard deviation (*n* = 5). *p < 0.05; **p < 0.01; one-way ANOVA. *P. aeruginosa*, *Pseudomonas aeruginosa*; MRSA, methicillin-resistant *Staphylococcus aureus*; VL, visible light; SA, sodium alginate; PNIPAM, poly(N-isopropylacrylamide); PTMH, programmed time-released multifunctional hydrogel; OD, optical density; DCFH-DA, 2′,7′-dichlorofluorescin diacetate; FSC, forward scatter; SSC, side scatter; ROS, reactive oxygen species; ANOVA, analysis of variance

In this study, the antibacterial effect of PTMH was assessed using two representative bacterial species, namely *Pseudomonas aeruginosa* (*P. aeruginosa*, ATCC 27,853) and MRSA, ATCC 43,300). The bacterial suspension was coincubated with ZnO@SA, Cu_5.4_O@PNIPAM, and PTMH with or without VL irradiation. As shown in Fig. 3A–C, ZnO@SA and PTMH groups with VL treatment exhibited a significantly reduced number of bacterial colonies formed by the coincubated bacterial suspension compared to that in the other groups, indicating that ZnO@SA and PTMH groups with VL treatment could efficiently inhibit the growth of *P. aeruginosa* and MRSA (*p* < 0.01). Additionally, the absorbance of the bacterial suspension at a wavelength of 600 nm, which serves as a standard metric for monitoring microbial density in liquid cultures, was significantly decreased (*p* < 0.01) following ZnO@SA and PTMHs treatment with VL (Fig. 3D–E). This trend exhibited a resemblance to the outcomes derived from the bacterial colony formation test. However, as previously shown by our research group [8], Cu_5.4_O@PNIPAM with or without VL treatment could not inhibit bacterial growth. Notably, compared with the groups without VL treatment, the number of bacterial colonies was significantly decreased in the groups (ZnO@SA and PTMH) with VL treatment (*p* < 0.01). According to previous research, this observation may be attributed to the photocatalytic effect of ZnO.

To further determine the underlying mechanism of the antibacterial effect of PTMH, we utilized a 2′,7′-dichlorofluorescin (DCFH) diacetate probe to measure the level of oxidative stress in live cells under different treatments. Following VL treatment, the green fluorescence intensity of cells from the ZnO@SA and PTMH groups was significantly increased compared with that of the other groups (Fig. 3F–G). Moreover, the percentage of DCF-positive cells under ZnO@SA and PTMH with VL treatment increased from 8.3 to 57.5% and 54.8% (*p* < 0.01), respectively (Fig. 3H–I). However, the percentage of DCF-positive cells in the groups without VL treatment remained low (9.14% and 4.93%, respectively). This finding demonstrated that PTMH could produce ROS through VL treatment and inhibit the growth of bacteria.

### Hemostatic ability assay

Hemorrhage resulting from physical injury, traumatic events, and surgical interventions constitutes a significant concern leading to morbidity and mortality [23]. Therefore, it is necessary to achieve hemostasis to promote tissue regeneration [24]. To examine the hemostatic ability, we conducted a coagulation test, as shown in Fig. S6A. In the absence of intervention, normal blood clots in 5–6 min. However, the as-prepared hydrogel could accelerate hemostasis (∼ 1 min, Fig. S6B). We further checked the clotting using SEM; there was plenty of fibrin in the clotting caused by our hydrogel compared with the control group (Fig. S7A). The size of the fibrin was quantified and is shown in Fig. S7B. The fibrin size in the hydrogel group was approximately 200 nm. However, the fibrin size in the control group was approximately 125 nm. In vivo hemostatic sealing of a rat liver was conducted to examine the hemostatic capability because less bleeding occurred in a cutaneous wound mouse model. The rapid hemostatic sealing of a bleeding rat liver in vivo by the hydrogel is shown in Fig. S8A. Following different treatments, blood loss was reduced in the as-prepared group (Fig. S8B). The time to hemostasis and blood loss until hemostasis is shown in Fig. S8C–D. Based on the above data, the as-prepared hydrogel could significantly reduce the blood clotting time. Bleeding is the first aspect that requires management in the process of wound healing [25]. Therefore, we added Ca^2+^ to enhance the hemostatic ability. The above results revealed that the prepared PTMHs possess good hemostatic ability.

### Promotion of impaired wound healing via PTMH

We further assess the effect of improving delayed wound repairing of PTMH. Diabetic foot ulcers pose a significant challenge in the management of individuals with diabetes due to their inherent vulnerability to bacterial infection, uncontrolled inflammation, and impaired angiogenesis, which impeded spontaneous healing [26, 27]. The high blood glucose and inhospitable environment surrounding the affected wound site frequently facilitates bacterial colonization [28].

**Fig. 4:**
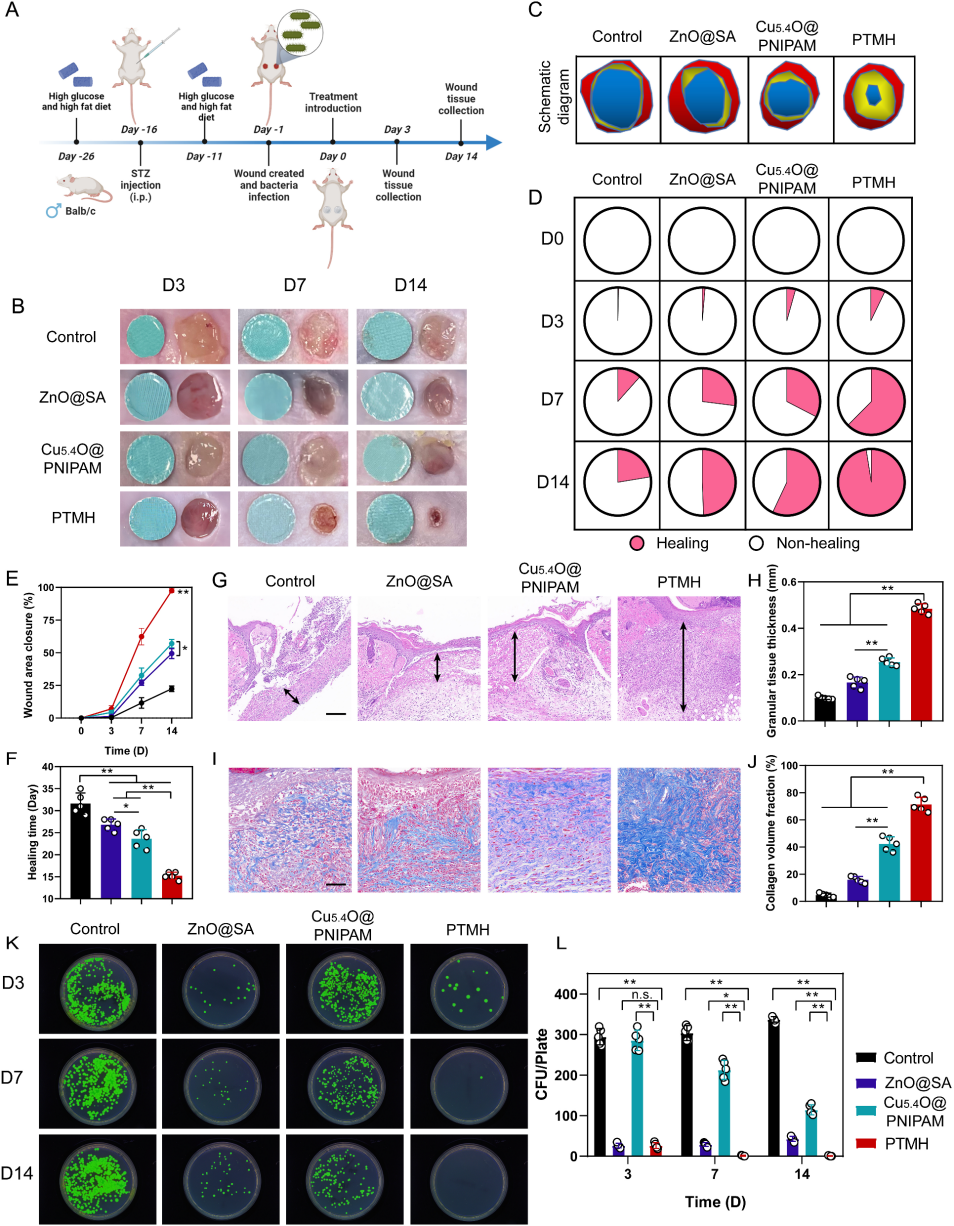
Efficiency of PTMHs for the healing of mouse diabetic wounds with *P. aeruginosa* infection. (A) Schematic of the experimental design for the therapeutic effect evaluation of PTMH in mouse STZ-induced diabetic wounds with *P. aeruginosa* infection. (B) Digital images of diabetic wounds at various time intervals, with a blue disc measuring 6 millimeters in diameter for scale reference. (C) Schematic images of diabetic wounds under different treatments. (D) The proportions of wound healing achieved through various treatment on days 0, 3, 7, and 14 (*n* = 5). (E) Area percentages of closed wounds. (F) Ultimate healing time of each group. (G) Representative histological images of H&E staining in the wound site on day 14. Black arrows represent the thickness of the granulation tissue. (Scale bar: 100 μm) (H) Statistical analysis of the thickness of the granulation tissue. (I) The images of Masson’s trichrome staining in diabetic wounds through various treatment. (Scale bar: 100 μm) (J) Statistical analysis of the percentage of collagen volume fraction. (K) Images and (L) quantitative counts of bacterial colonies formed by *P. aeruginosa* harvested from wound tissues at various time intervals. Data in E, F, H, J, and L represent the mean ± standard deviation (*n* = 5). *p < 0.05; **p < 0.01; n.s., no significance; one-way ANOVA. SA, sodium alginate; PNIPAM, poly(N-isopropylacrylamide); PTMH, programmed time-released multifunctional hydrogel; *P. aeruginosa*, *Pseudomonas aeruginosa*; STZ, streptozotocin; ANOVA, analysis of variance; CFU, colony-forming unit; H&E, hematoxylin and eosin

The infection models were established one day following the introduction of *P. aeruginosa* into the wounds that were induced on the dorsal region of streptozotocin-induced diabetic mice (Fig. 4A) [29]. The model mice were randomly divided into four groups: (1) Control group (treated with PBS); (2) ZnO@SA group (with VL irradiation); (3) Cu_5.4_O@PNIPAM group (with VL irradiation); and (4) PTMH group (ZnO@SA-Cu_5.4_O@PNIPAM PTMH with VL treatment). The adhesion achieved by amine groups on chitosan are responsive to changes in pH. When pH < 6.5, [NH_2_] < [NH_3_^+^], and the chitosan chains dissolve in water as a polyelectrolyte. When pH > 6.5, [NH_2_] > [NH_3_^+^], and the NH_2_-OH hydrogen bond promotes the chitosan chains to form a network [30]. The wounds from each group were photographed on days 0, 3, 7, and 14 post-treatment. The wounds treated with PTMH showed obviously accelerated wound closure (*p* < 0.01) (Fig. 4B–C and Fig. S9). Quantitatively, the PTMH group led to 62.4% and 97.48% wound closure on days 7 and 14 post-treatment, respectively. These rates were markedly higher (*p* < 0.01) than those of the PBS-treated wounds (11.6% and 22.4%, respectively) (Fig. 4D–E). On day 3 post-treatment, PTMHs led to less inflammatory exudation with less infection status compared with control (*p* < 0.01), which may be attributed to the antibacterial effect of ZnO. Additionally, five mice from each group were fed continuously until the wounds completely healed. As shown in Fig. 4F, the wounds of mice treated with PTMHs had healed 15.2 days post-treatment and were significantly shorter (*p* < 0.01) than those of mice treated with PBS, ZnO@SA, and Cu_5.4_O@PNIPAM (31.6, 25, and 23.6 days, respectively). Notably, Cu_5.4_O@PNIPAM showed a greater wound-healing promotion effect than ZnO@SA, which was attributed to the antioxidative effect of Cu_5.4_O nanoparticles and the cutaneous contraction effect of PNIPAM hydrogels.

Histological analysis was employed to investigate the wound healing processes in further detail, utilizing techniques, such as H&E and Masson’s trichrome staining methods [31]. Among all groups, the wounds in the PTMH treatment group exhibited the thickest granulation tissue (*p* < 0.01) according to H&E section images (Fig. 4G–H). Moreover, we evaluated the collagen content at the diabetic wound sites using Masson’s trichrome staining. As shown in Fig. 4I–J, wounds treated with ZnO@SA, Cu_5.4_O@PNIPAM, and PTMH showed higher collagen content than the control group (*p* < 0.01), whereas the PTMH group showed more than the ZnO@SA and Cu_5.4_O@PNIPAM groups (*p* < 0.01). It demonstrated that the development of granulation tissue and collagen was significant improved after 14 days of PTMH treatment, which showed an accelerated wound healing.

The antibacterial performance of PTMH was evaluated by collecting and homogenizing the mouse cutaneous tissue in wound areas on days 3, 7, and 14 (Fig. 4K–L). On day 3, a lower number of bacterial colonies was observed in the ZnO@SA and PTMH groups (*p* < 0.01). However, the number of bacterial colonies from the PTMH groups on days 7 (*p* < 0.05) and 14 (*p* < 0.01) was significantly lower than that of the ZnO@SA groups, indicating that ZnO@SA could inhibit bacterial growth in the short term, and that PTMH could release Cu_5.4_O nanoparticles from day 3 onward, thereby improving the microenvironment of wound tissue in the middle and late stages of wound healing. Notably, PTMHs were associated with the lowest number of bacterial colonies (*p* < 0.01) during the entire period of the experiment due to their antibacterial effect of ZnO and anti-inflammatory effect of Cu_5.4_O, indicating long-term effective inhibition of bacteria. Patients with diabetic wound are susceptible to bacterial infection due to the microenvironment of high blood glucose and extensive inflammation, which hinders the normal process of wound healing [32, 33]. Thus, the efficient antibacterial activities of PTMHs provided a sterile microenvironment for subsequent repair stages.

**Fig. 5:**
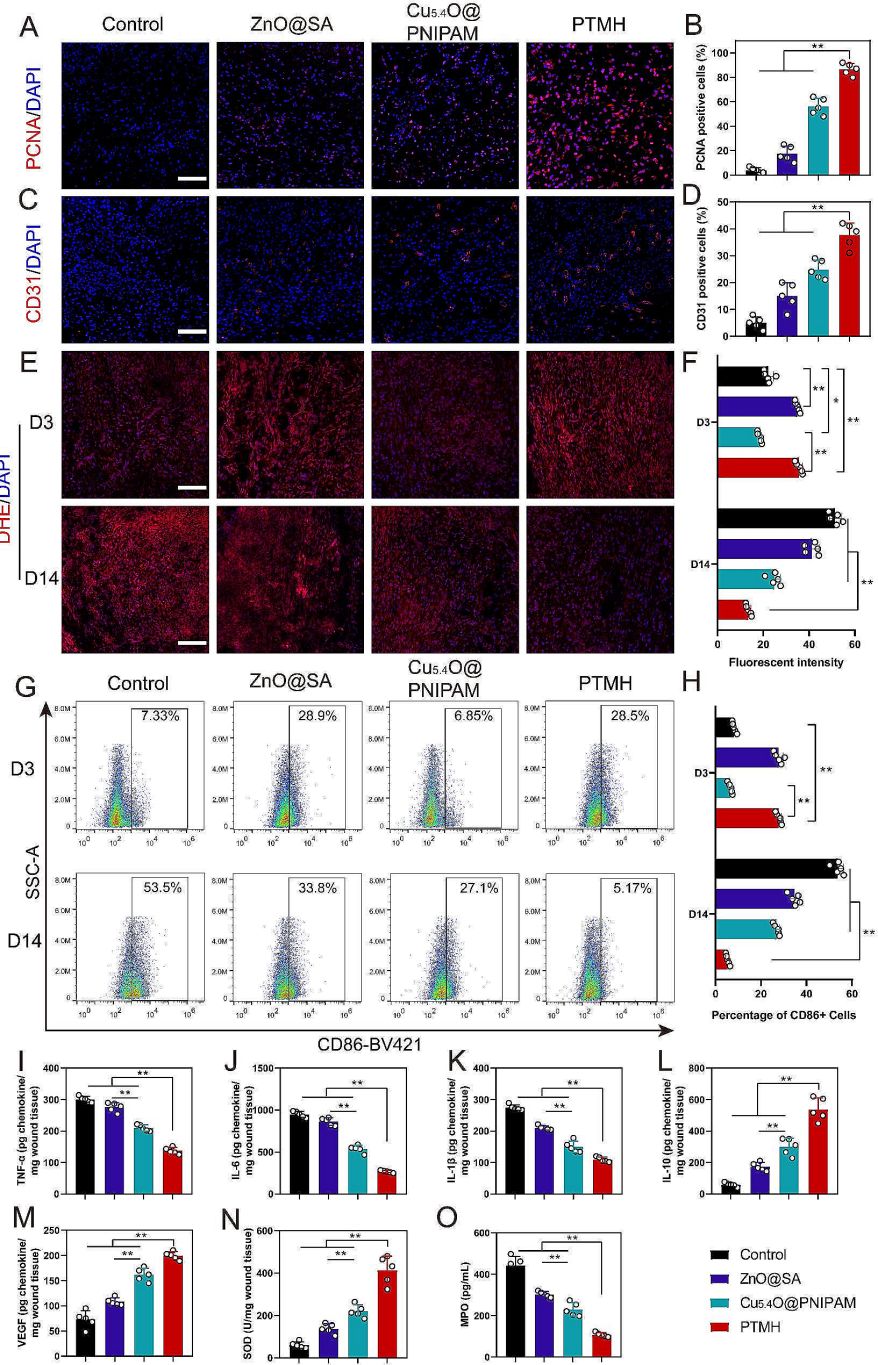
Regulatory effects of PTMH on the microenvironment in vivo. (A) Fluorescent images and (B) statistical analysis of PCNA (red) and DAPI (blue) in mouse diabetic wounds tissue. (Scale bar: 100 μm) (C) Fluorescent images and (D) statistical analysis of CD31 (red) and DAPI (blue) in mouse diabetic wounds tissue. (Scale bar: 100 μm) (E) Fluorescent images and (F) statistical analysis of DHE (red) and DAPI (blue) on days 3 and 14 post-treatment wound tissue. (G) The distribution of cells in wound tissue obtained from different groups on days 3 and 14 post-treatment, stained with rat anti-mouse CD11b-Brilliant Violet 650™, LY6G-FITC, and CD86-Brilliant Violet 421™ (1:200). (H) Statistical analysis of CD86-Brilliant Violet 421^TM^-positive cells. (I) The concentrations of TNF-α, (J) IL-6, (K) IL-1β, (L) IL-10, (M) VEGF, (N) SOD and (O) MPO in wound tissue were determined using ELISA. Data in B, D, F, H and I–O represent the mean ± standard deviation (*n* = 5). *p < 0.05; **p < 0.01; one-way ANOVA. SA, sodium alginate; PNIPAM, poly(N-isopropylacrylamide); PTMH, programmed time-released multifunctional hydrogel; PCNA, proliferation cell nuclear antigen; DHE, dihydroethidium; CD, cluster of differentiation; ELISA, enzyme-linked immunosorbent assay; TNF-α, tumor necrosis factor-α; IL, interleukin; VEGF, vascular endothelial growth factor; SOD, superoxide dismutase; MPO, myeloperoxidase; ANOVA, analysis of variance; DAPI, 4’,6-diamidino-2-phenylindole; SSC-A, side scatter area

To further determine the effect of PTMH on wound healing promotion by fluorescent staining, wound tissue from day 14 post-treatment was harvested, sectioned, and stained with the corresponding antibodies. PCNA, as a marker of proliferation, was selected for the detection of tissue proliferation under different treatments [34]. The highest number of red fluorescence-positive cells was observed in the PTMH group (*p* < 0.01), indicating the ability of PTMH to promote tissue repair (Fig. 5A–B). In previous studies [8], we demonstrated that Cu_5.4_O nanoparticles effectively stimulated the proliferation and migration of vascular endothelial cells, thereby contributing to the promotion of wound healing. Consequently, it is of great interest to assess the in vivo angiogenic impacts of PTMHs. Blood vessels transport essential nutrients to the site of injury, thereby facilitating the process of wound healing. As shown in Fig. 5C–D, the number of blood vessels was significantly increased in the Cu_5.4_O@PNIPAM-treated wounds (*p* < 0.01) and PTMH-treated wounds (*p* < 0.01) compared with that in the PBS-treated wounds, indicating that PTMH exert angiogenic effects in vivo.

Furthermore, in order to detect the level of reactive oxygen species (ROS) at the site of the wound, the tissue surrounding the wound was collected on days 3 and 14 after the surgical procedure. Subsequently, the collected tissue was promptly frozen using liquid nitrogen to generate frozen sections, which were then subjected to staining with the fluorescent dye dihydroethidium (DHE) indicating the ROS level of tissues [35]. As shown in Fig. 5E–F, on day 3, the fluorescence intensities were significantly higher for ZnO@SA (*p* < 0.05) and PTMHs (*p* < 0.01) compared with those for Cu_5.4_O@PNIPAM due to the production of oxygen free radicals by ZnO with VL irradiation. However, on day 14, the fluorescence intensities for the PTMHs were apparently decreased compared with those of the other three groups. This may be attributed to the antioxidative effect of Cu_5.4_O. To further confirm this hypothesis, M1 macrophages in wound tissue were quantified using FACS [13]. M1 macrophages can be activated by a series of inflammatory factors strongly associated with ROS and further produce ROS to enhance inflammation [36]. In contrast, M1 macrophages are involved in the immune system response of mice towards invasive microorganisms, such as bacteria [37]. On day 3, the number of CD86-positive cells (M1 macrophages) was significantly increased in the ZnO@SA (*p* < 0.05) and PTMH (*p* < 0.01) groups compared with the control and Cu_5.4_O@PNIPAM groups (Fig. 5G–H and S10). Conversely, on day 14, the number of CD86-positive cells (M1 macrophages) was significantly decreased in the PTMH group, whereas it was the highest in the control group. This trend was consistent with the results obtained from DHE staining images, confirming that on day 3, M1 macrophages could be activated by ROS produced through ZnO with VL irradiation, which contributed to alleviating the bacterial infection and activating the subsequent signaling pathway for repair promotion. In contrast, on day 14, the release of Cu_5.4_O inhibited the excessive ROS production and inflammation, promoted the microenvironment, and ultimately enhanced wound healing.

Moreover, proinflammatory factors, as a significant medium, maintained chronic and high-level inflammation in diabetic wounds [38]. Corresponding ELISA kits were used to detect the concentration of proinflammatory factors in wound tissue collected on day 14 post-treatment. As shown in Fig. 5I–K, the concentrations of TNF-α, IL-6, and IL-1β in wound tissue were dramatically decreased following treatment with PTMH (*p* < 0.01), whereas those of IL-10 (a classic anti-inflammatory factor) were significantly increased compared with those of the other groups (*p* < 0.01) (Fig. 5I). This demonstrated that PTMH could obviously inhibit inflammation in the wound repairing stage. Vascular endothelial growth factor (VEGF), a cytokine that promotes endothelial cell proliferation, is easily inactivated under excessive oxidative stress and inflammation, thereby inhibiting the process of angiogenesis [39]. Notably, the concentration of VEGF was significantly increased in the PTMH treatment group compared with the other groups (*p* < 0.01) (Fig. 5M). This trend was consistent with the results obtained from CD31 staining (Fig. 5C).

In addition, superoxide dismutase (SOD) and myeloperoxidase (MPO) were selected as determinants of oxidative stress in wound tissue. SOD, an enzyme with antioxidant properties, is capable of scavenging ROS to uphold a harmonized redox equilibrium within biological systems. SOD demonstrates its efficacy in assessing the degree of oxidative stress and hypoxia by impeding lipid peroxidation within wound tissue and prompting tailored reactions to inflammatory stimuli [40]. It is important to acknowledge that diminished SOD activity can intensify the oxidative harm linked to diabetes [8]. In comparison to the control group, the PTMH treatment group exhibited a sustained elevation in SOD activity (*p* < 0.01) (Fig. 5N). The glycosylated enzyme MPO is found in the granules of neutrophils and macrophages, playing a vital role in promoting inflammatory injuries caused by oxidative stress and serving as a biomarker for monitoring the advancement of diabetic wounds. Consequently, assessing MPO activity enables the evaluation of immune cell infiltration, including neutrophils and M1 macrophages, in inflamed tissue [14]. Remarkably, the control group demonstrated a noteworthy elevation in MPO activity, which was significantly reduced with PTMH treatment (Fig. 5O). It suggested that the administration of PTMH treatment effectively suppresses oxidative stress within inflamed wound tissue.

Our results of diabetic wound healing showed that there is little difference between ZnO and Cu_5.4_O groups. On the one hand, ZnO could eliminate bacteria by producing ROS, which could efficiently control the status of bacterial infection in the early stage of wound healing. However, the process of diabetic wound healing was impeded by not only bacterial infection but uncontrolled inflammation. In the stage of inflammation, excessive ROS and proinflammatory factors impaired the function of fibroblasts and vascular endothelial cells. Consequently, the healing process was stalled in the inflammatory phase. On the other hand, Cu_5.4_O nanoenzyme could scavenge ROS and inhibit the level of inflammation, which could regulate inflammation in wound tissue and improve the effect of angiogenesis. However, moderate ROS could inhibit the growth of bacteria and simply using Cu_5.4_O nanoenzyme in the early stage of wound healing may aggravate infection. ZnO and Cu_5.4_O can complement each other to address their respective limitations and capitalize on their advantages. ZnO efficiently eliminated bacteria in the early phase of healing and Cu_5.4_O scavenged excessive ROS in the stage of inflammation, thereby achieving the improvement of microenvironment of diabetic infected wound.

### Therapeutic mechanisms of PTMH in impaired wound healing

**Fig. 6:**
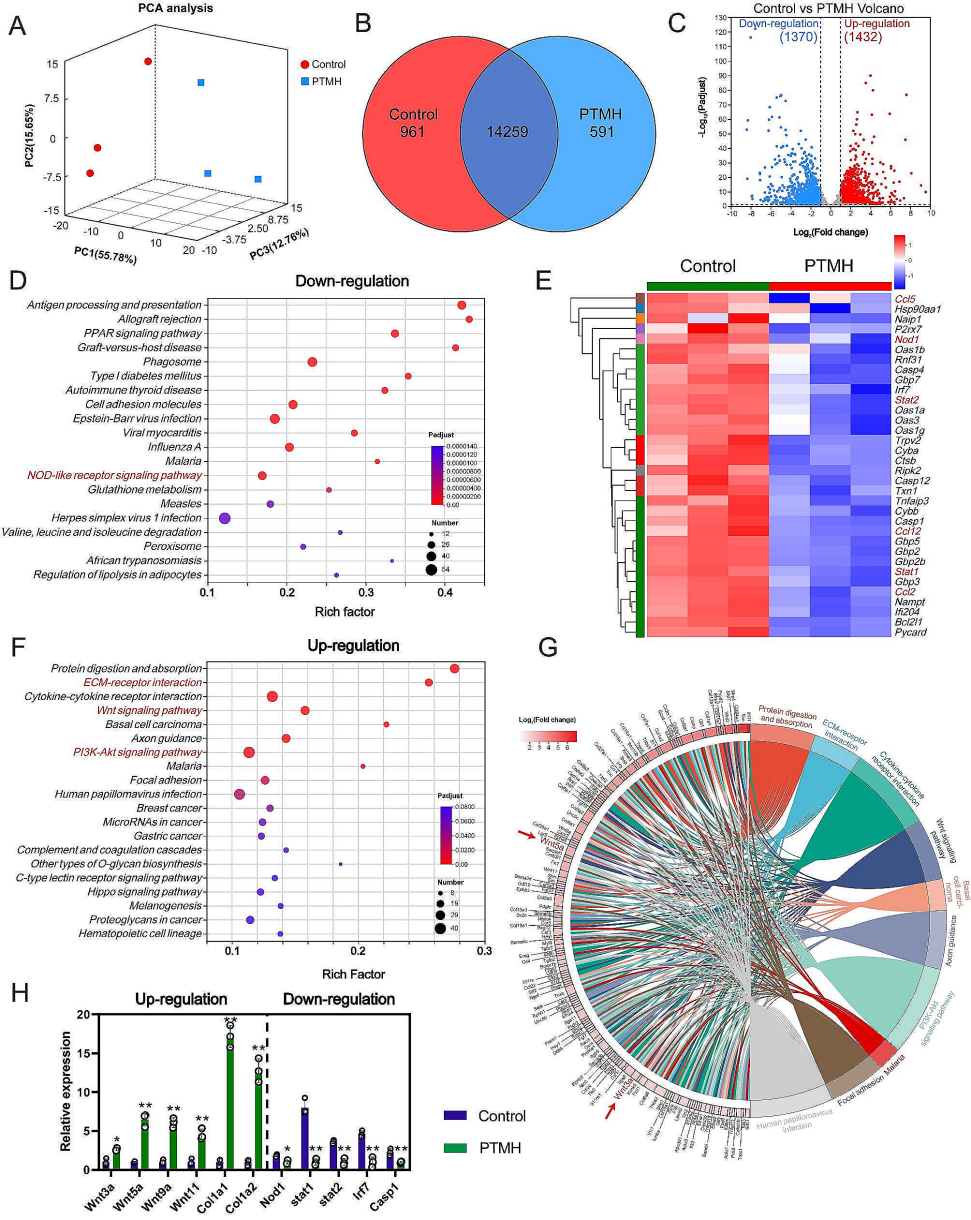
Mechanisms underlying the therapeutic effects of PTMH in impaired wound healing. (A) Principal Component Analysis (PCA) was performed on the differentially expressed genes in the wound tissues of the mice subjected to treatment with PTMH (PTMH group) or PBS (Control group). Each data point within the two groups represents an independent replicate. (B) Venn diagram was constructed based on the transcriptomic data to illustrate the overlap between the two groups. (C) Volcano plots were generated to visualize the genes that were upregulated and downregulated after PTMH treatment. (D) KEGG pathway enrichment analysis of the differentially expressed genes (downregulation). (E) Heatmaps illustrating the noteworthy downregulation of genes implicated in inflammation and oxidative stress subsequent to PTMH treatment. (F) KEGG pathway enrichment analysis of the differentially expressed genes (upregulated). (G) Chord diagram showing the genes involved in inflammation and oxidative stress that were significantly upregulated following PTMH treatment. (H) Relative expression of genes involved in inflammation and wound regeneration. Data in h represent the mean ± standard deviation from three independent replicates (*n* = 3). *p < 0.05; **p < 0.01; n.s., no significance; *t-*test. PTMH, programmed time-released multifunctional hydrogel; PCA, principal component analysis; IL, interleukin; KEGG, Kyoto Encyclopedia of Genes and Genomes; ECM, extracellular matrix; Nod1, nucleotide-binding oligomerization domain containing 1

In order to investigate the underlying mechanism through which PTMH promote wound healing, transcriptome sequencing was utilized to perform quantitative RNA analysis. According to unguided principal component analysis (PCA) of the data, the transcriptomics profiles of cutaneous wounds in diabetic mice treated with PTMH and wound dressing exhibited significant differences (Fig. 6A). Based on the Venn diagram, 14,259 genes were shared by the PTMH treatment and control groups, 961 and 591 genes were unique to the PTMH and control groups, respectively (Fig. 6B). Furthermore, volcano plots revealed significant differential expression of RNAs (*p* < 0.05). Among these, 1,370 RNAs were found to be downregulated as determined by a cutoff value of 0.83-fold change, while 1,432 RNAs were upregulated as determined by a cutoff value of 1.2-fold change, following PTMH treatment (Fig. 6C).

Gene ontology (GO) enrichment analysis was performed to determine the enrichment pathways of differentially expressed genes (DEGs) that were downregulated. Following PTMH treatment, downregulated DEGs were significantly enriched in inflammation-related pathways, such as “positive regulation of IL-1β production” (Fig. S11). As shown in Fig. 6D and S12, the KEGG pathway enrichment analysis showed that the downregulated DEGs in the PTMH treatment group were involved in the nucleotide-binding oligomerization domain (NOD)-like receptor (NLR) signaling pathways associated with inflammation and oxidative stress in cutaneous wounds [41]. Among these downregulated genes, inflammation-associated genes, such as NOD-containing protein 1 (Nod1), signal transducer and activator of transcription 1 (Stat1), Stat2, C-C motif chemokine ligand 2 (Ccl2), Ccl5, and Ccl12, were significantly inhibited following PTMH treatment (Fig. 6E and H). We constructed a protein‒protein interaction (PPI) network comprising key downregulated genes, suggesting that the interaction of these key genes is related to inflammation and oxidative stress (Fig. S13). A subfamily of NLR signaling pathways is primarily involved in the activation of inflammation, which has been implicated in a multitude of disease models and human diseases [41]. Bauernfeind et al. demonstrated that increased ROS levels promote NLR family pyrin domain containing 3 (NLRP3) expression at the transcriptional level [42]. NLRP3 could lead to the maturation of proinflammatory factors [42], such as IL-1β and IL-18, and the decreased concentration of IL-1β following PTMH treatment (Fig. 5K) also verified this trend. The dysregulation and hyperactivation of the NLR signaling pathway are closely related to excessive inflammation and oxidative stress, which may impede the process of diabetic wound healing. Following PTMH treatment, the NLR signaling pathway was significantly downregulated, and this effect may be attributed to two reasons. First, PTMH exerted a strong antibacterial effect, particularly in the first three days postinjury, which controlled the infection and improved the microenvironment of wound healing at the early stage. Second, the NLR signaling pathway may be downregulated via the antioxidative and anti-inflammatory effect of Cu_5.4_O during the repair stage. By the synergistic effect in time-sequence, the inflammation-associated pathway would be downregulated, and the microenvironment of wound tissue would be improved.

GO and KEGG analyses were performed to further determine the enrichment of upregulated DEGs. The results of GO analysis showed that DEGs were enriched in the “noncanonical Wnt signaling pathway” and “extracellular matrix assembly” (Fig. S14). The results of KEGG analysis demonstrated that the DEGs upregulated following PTMH treatment were involved in “extracellular matrix–receptor interaction” (Fig. 6F–G and S15). Furthermore, the KEGG analysis indicated that the key enrichment pathway is mainly centered on the Wnt signaling pathway (Fig. 6F–G and S16). The heatmaps and PPI networks also showed that regeneration-related genes, such as Wnt 3a and Wnt 5a, were significantly upregulated (Fig. 6H and S17–18). The upregulated Wnt signaling pathway could facilitate the proliferation of vascular endothelial cells and promote angiogenesis [43]. The promotion of accelerated angiogenesis plays a critical role in the process of healing diabetic wounds, as it facilitates the delivery of oxygen and nutrients to the compromised tissue, thereby mitigating extensive inflammation and combating bacterial infection [44]. Nevertheless, the presence of an extensive local inflammatory reaction hinders the progression of angiogenesis in diabetic wounds, including bacterial infection, excessive oxidative stress, and uncontrolled infiltration of inflammatory cells. Excess ROS production may inactivate cytokines, such as VEGF, and cause vascular endothelial cell apoptosis, which may dramatically impede the process of angiogenesis and wound healing. As we previously described [8], Cu_5.4_O ultrasmall nanoparticles exhibit remarkable antioxidant efficiency both in vitro and in vivo and promote the proliferation of vascular endothelial cells. In the present study (Fig. 5C, D, and M), the number of blood vessels and the concentration of VEGF in the PTMH treatment group were significantly increased, demonstrating the improvement of angiogenesis, which was consistent with the results of the upregulation of Wnt signaling.

In summary, we have developed a PTMH system that can combat bacterial infection, inhibit inflammation, and promote impaired wound healing in a time-dependent manner according to the characteristics of the different stages of the wound healing process. Hence, forthcoming versions of this platform, in conjunction with our PTMHs, have the potential to be programmed for the generation of diverse immunotherapeutic and other substances, facilitating the evaluation of various rational therapeutic combinations. Additionally, we are exploring a real-time monitoring system for the detection of inflammatory cell infiltration and the dynamic assessment of oxidative stress levels, which would enhance the precision and efficiency of this therapeutic system. Finally, identification of better drug-loading options in additional translational animal models is imperative to improve the clinical relevance of this system.

### Biocompatibility of PTMH in vivo

The biocompatibility of PTMH was assessed by examining the organ histopathology and blood chemistry of healthy mice. As shown in Fig. S19, there were no signs of necrosis, congestion, or hemorrhage observed in heart, liver, spleen, lung, and kidney tissues at the 3- and 30-day intervals, following treatment with PTMH. The comprehensive analysis of the blood panel (Fig. S20A–O) did not indicate a significant difference in the hematology between the PTMH treatment and control groups (*p* > 0.05). Furthermore, the examination of serum biochemistry (Fig. S20P-S) revealed that the levels of liver function markers aspartate aminotransferase and alanine aminotransferase, as well as kidney function markers blood urea nitrogen and creatinine, were comparable between the treatment and control groups (*p* > 0.05). This evidence further demonstrated the excellent biocompatibility of PTMH.

## Conclusions

We constructed a PTMH system to effectively combat bacterial infection and control the inflammation at wound sites in a sequential manner, thereby promoting impaired wound healing. The PTMH were designed to release ZnO, which effectively inhibits bacterial growth and establishes a sterile microenvironment for subsequent repair stages. Following a 3-day antibacterial treatment, the PTMH system additionally released Cu_5.4_O to mitigate oxidative stress and inflammation in wound tissue during the repair phase. In summary, this therapeutic system was designed to administer drugs in a time-sequence based on the distinct characteristics exhibited during various stages of the wound healing process. Consequently, our research outcomes present an innovative therapeutic approach for addressing conditions resulting from multifactor-associated inflammation.

### Credit author statement

Yuan Peng: Methodology, Validation, Investigation, Writing-original draft. Yicheng Guo: Methodology, Validation, Investigation, Formal analysis, Writing-original draft. Xin Ge: Methodology, Validation, Investigation, Formal analysis. Yali Gong: Methodology, Software. Yuhan Wang: Methodology, Software. Zelin Ou: Methodology. Gaoxing Luo: Supervision, Funding acquisition. Rixing Zhan: Conceptualization, Methodology, Project administration, Funding acquisition. Yixin Zhang: Conceptualization, Methodology, Project administration, Funding acquisition.

**Fig. 7:**
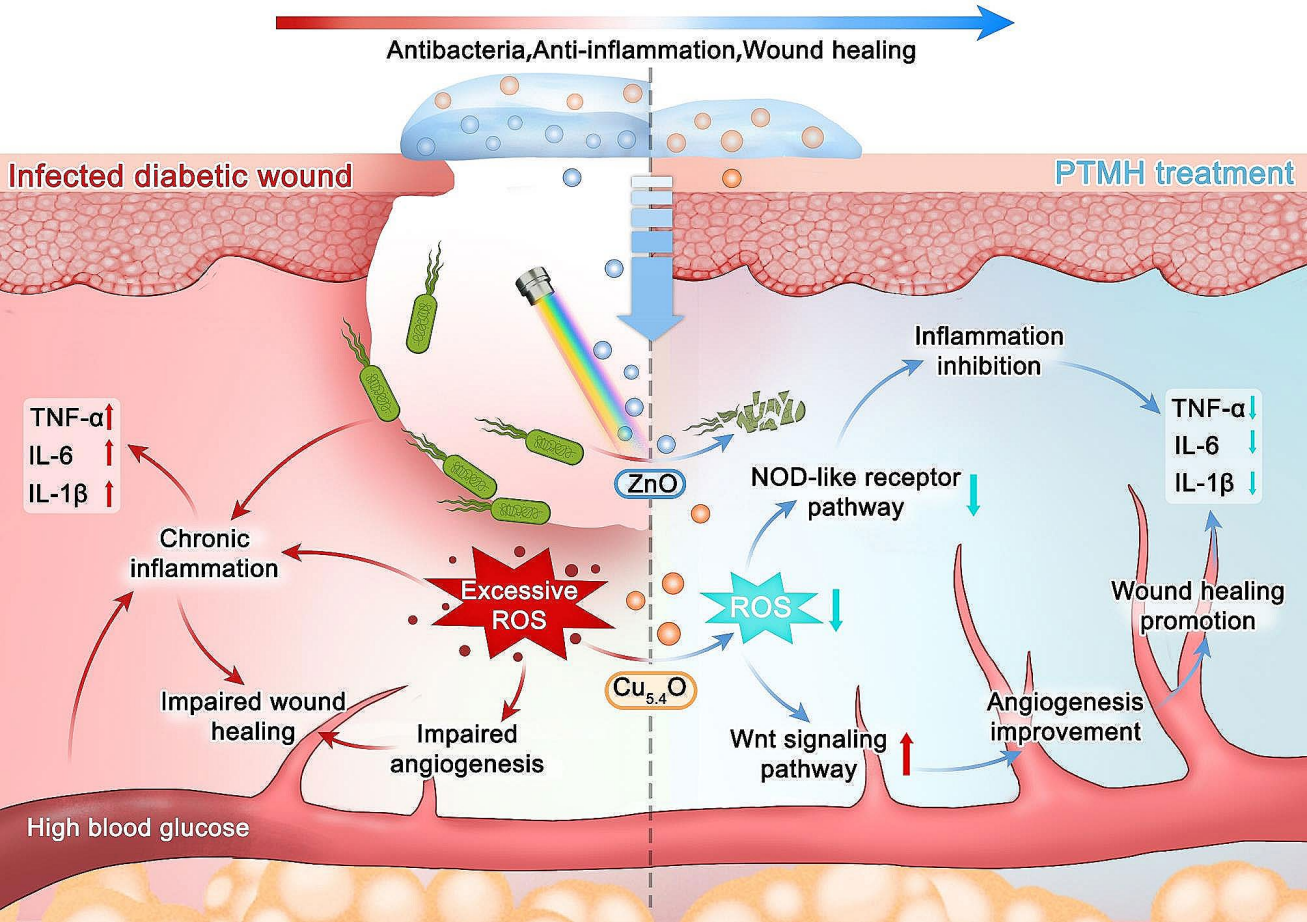
Schematic of the underlying mechanism of the effects of PTMH in impaired wounds healing. PTMH could combat bacterial infection by ZnO and alleviate inflammation via Cu_5.4_O ultrasmall nanozymes to improve the tissue repairing microenvironment, and ultimately promote impaired wound healing. IL, interleukin; PTMH, programmed time-released multifunctional hydrogel; ROS, reactive oxygen species; TNF-α, tumor necrosis factor-α

### Electronic supplementary material

Below is the link to the electronic supplementary material.

Supplementary Material 1

## Data Availability

No datasets were generated or analysed during the current study.
