# Supplementary material for: Construction of programmed time-released multifunctional hydrogel with antibacterial and anti-inflammatory properties for impaired wound healing
Source: J Nanobiotechnology. 2024 Mar 23;22:126. doi: 10.1186/s12951-024-02390-y (PMC10960406; doi:10.1186/s12951-024-02390-y)
Supplement: Supplementary file 1 — Supplementary Material 1 [file 12951_2024_2390_MOESM1_ESM.docx]

**Construction of Programmed Time-released Multifunctional Hydrogel with** **Antibacterial and Anti-inflammatory Properties for Impaired Wound Healing**

Yuan Peng^1#^, Yicheng Guo^2#^, Xin Ge^3#^, Yali Gong^2^, Yuhan Wang^2^, Zelin Ou^2^, Gaoxing Luo^2*^, Rixing Zhan^2*^, Yixin Zhang^1*^

1. Department of Plastic and Reconstructive Surgery, Shanghai Ninth People’s Hospital, Shanghai JiaoTong University School of Medicine, 639 Zhi Zao Ju Road, Shanghai 200011, China

2. Institute of Burn Research, Southwest Hospital, State Key Lab of Trauma, Burn and Combined Injury, Chongqing Key Laboratory for Disease Proteomics, Third Military Medical University (Army Medical University), Chongqing 400038, China

3. Department of Gastroenterology, The Second Affiliated Hospital of Chongqing Medical University, Chongqing 400010, China

Email: logxw@hotmail.com (G.L.); zhanrixing@sina.com (R.Z.); zhangyixin6688@163.com (Y.Z.)

# These authors contributed equally to this work.

* Corresponding author.


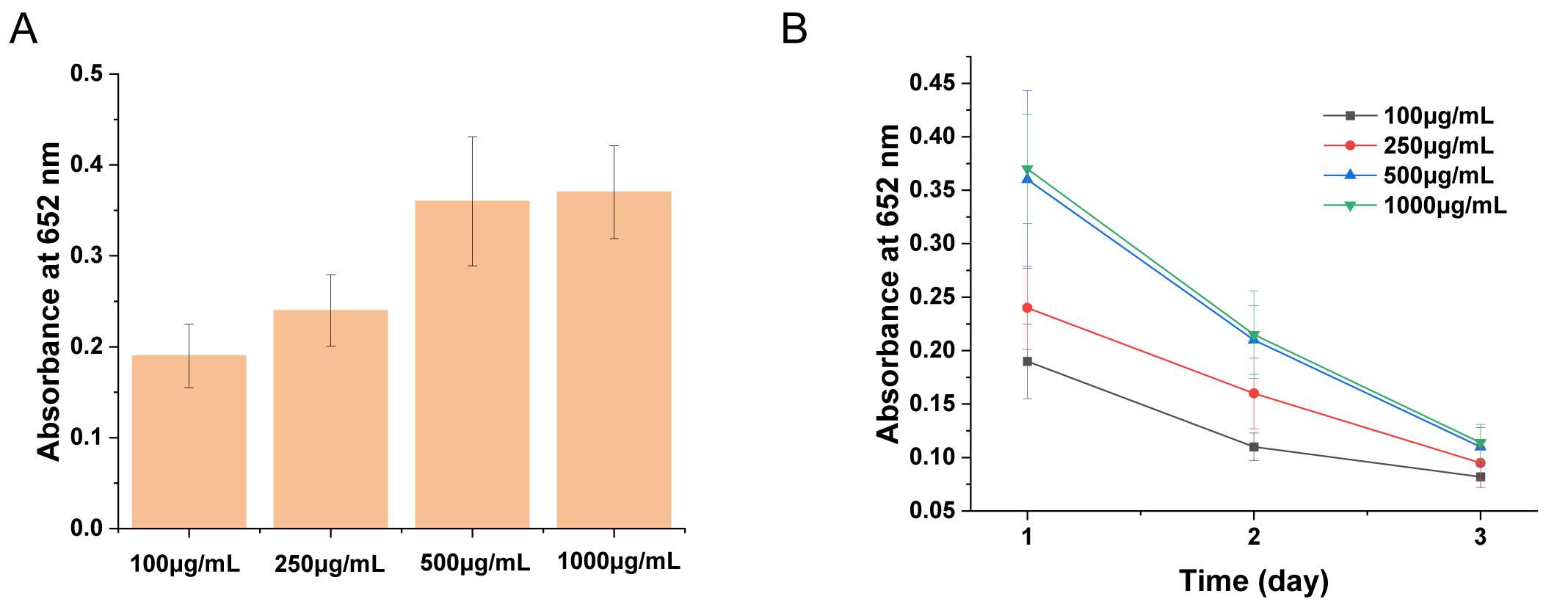


**Figure S1.** (A) The test of ROS production of ZnO (n = 5). (B) The level of ROS generated by ZnO in 3 days (n = 5).


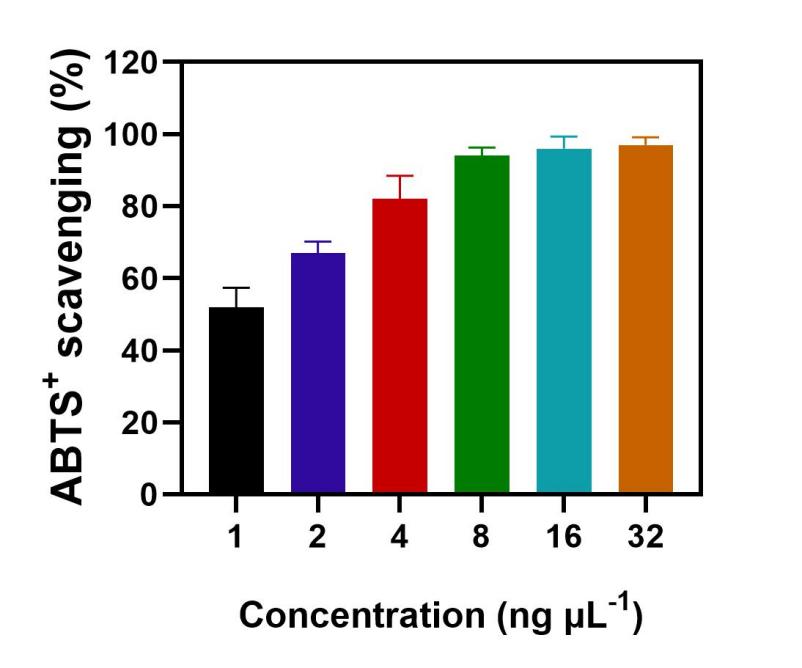


**Figure S2.** ABTS radical scavenging ratio of against different concentrations of Cu_5.4_O@CS (n = 5).


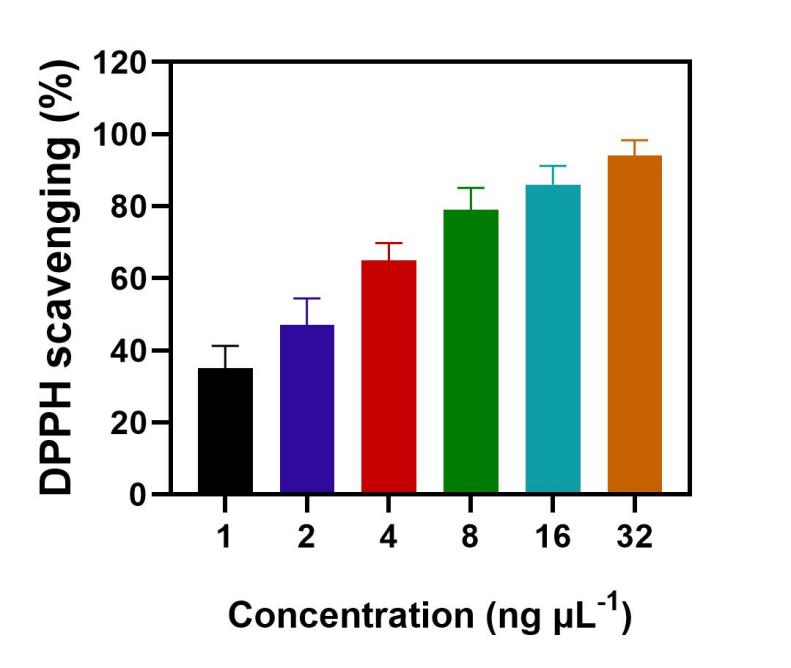


**Figure S3.** DPPH radical scavenging ratio of against different concentrations of Cu_5.4_O@CS (n = 5).


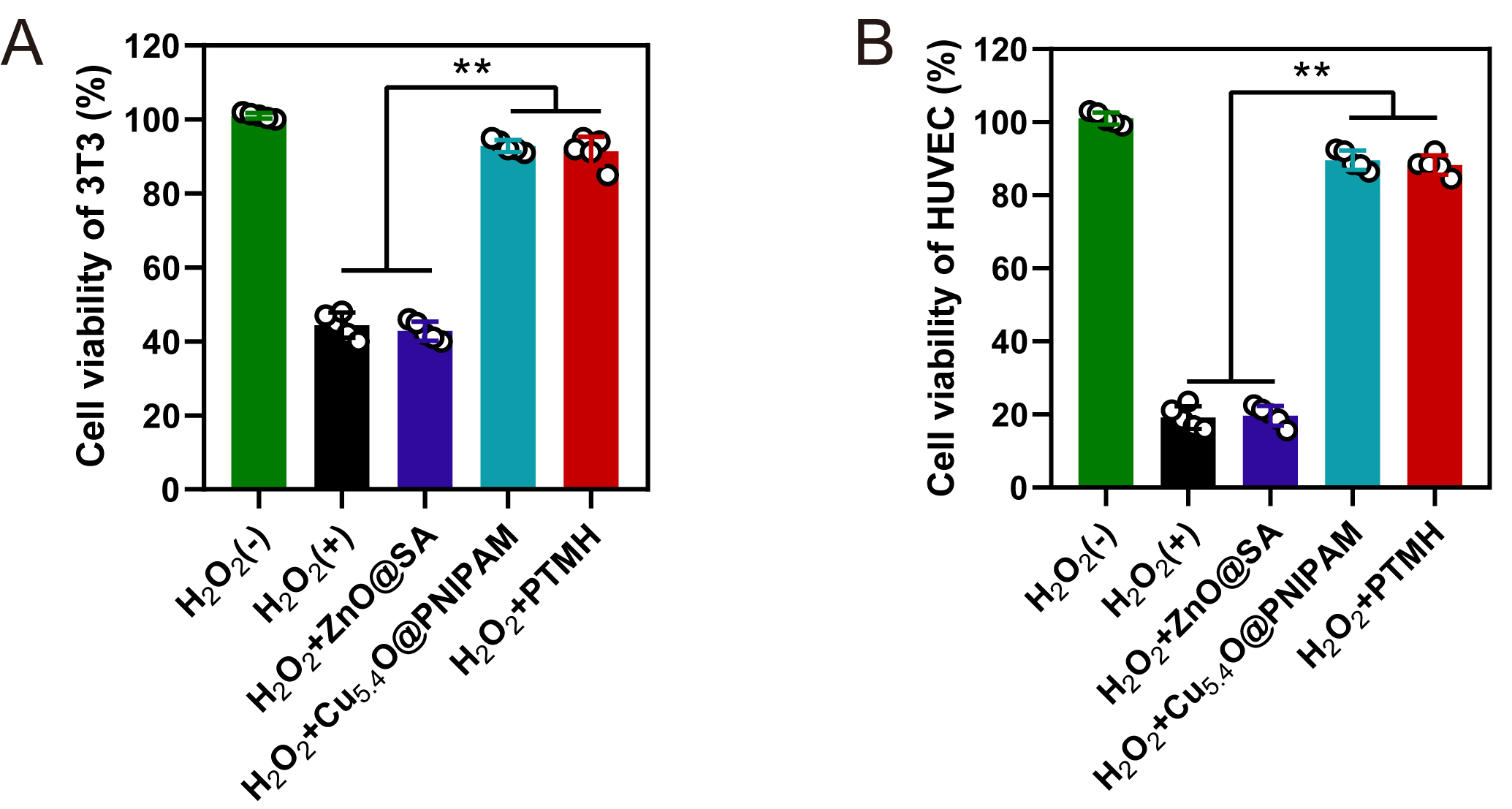


**Figure S4.** (A) The cell viability of 3T3 cells and (B) HUVEC cells under different treatment. Data in A and B represent the mean ± standard deviation from five independent replicates (n = 5). ***p* < 0.01, One-way ANOVA.


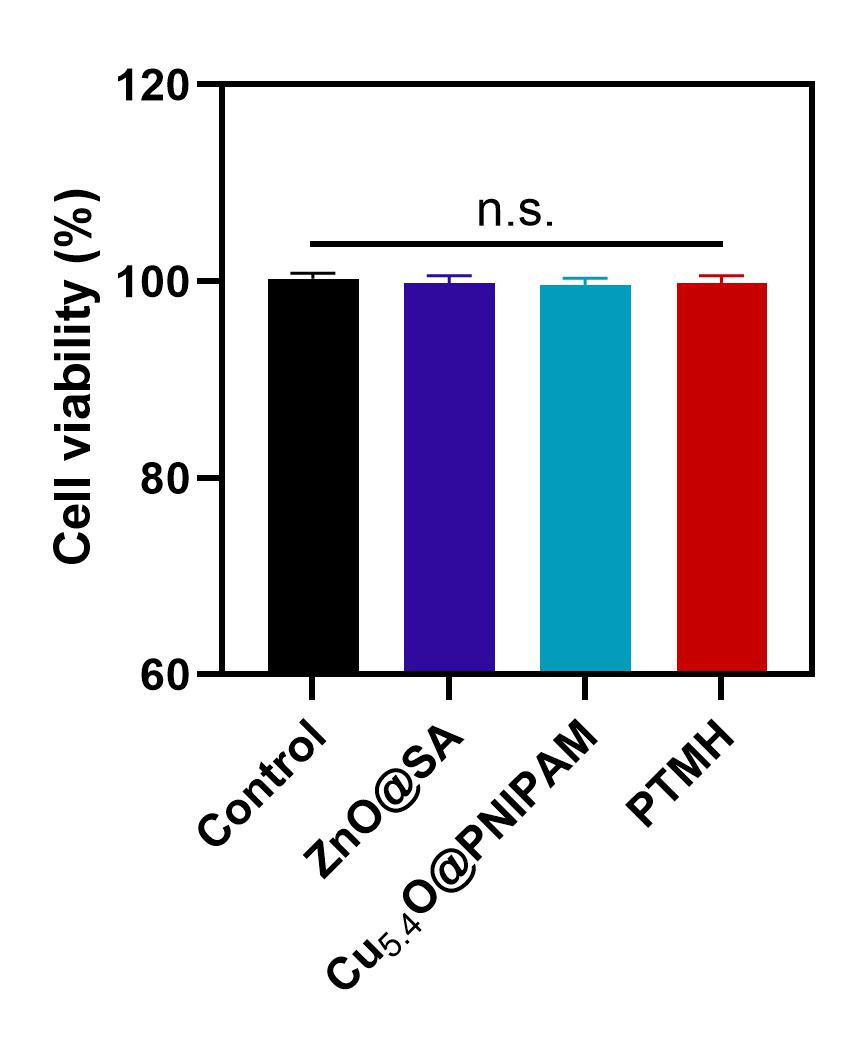


**Figure S5.** The cell viability of 3T3 under different treatment (n = 5). n.s., no significance, One-way ANOVA.


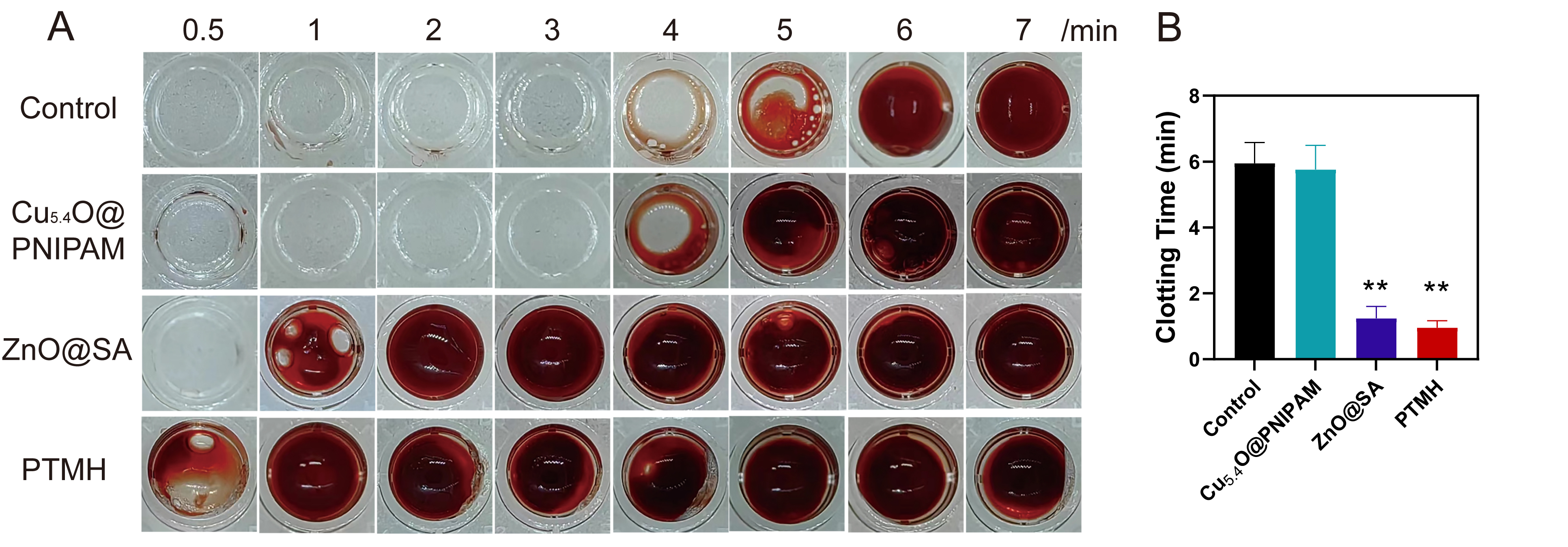


**Figure S6.** (A) Representative images of clotting formation. (B) Quantitative clotting time. Data in B represent the mean ± standard deviation from five independent replicates (n = 5). ***p* < 0.01, One-way ANOVA.


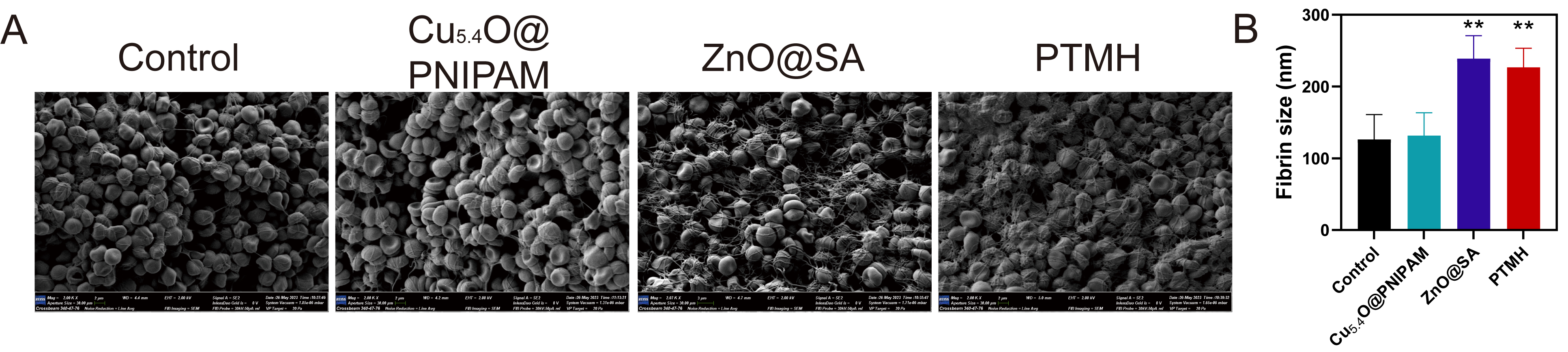


**Figure S7.** (A) Representative SEM images of whole-blood. (B) Quantitative fibrin size. Data in B represent the mean ± standard deviation from five independent replicates (n = 5). ***p* < 0.01, One-way ANOVA.


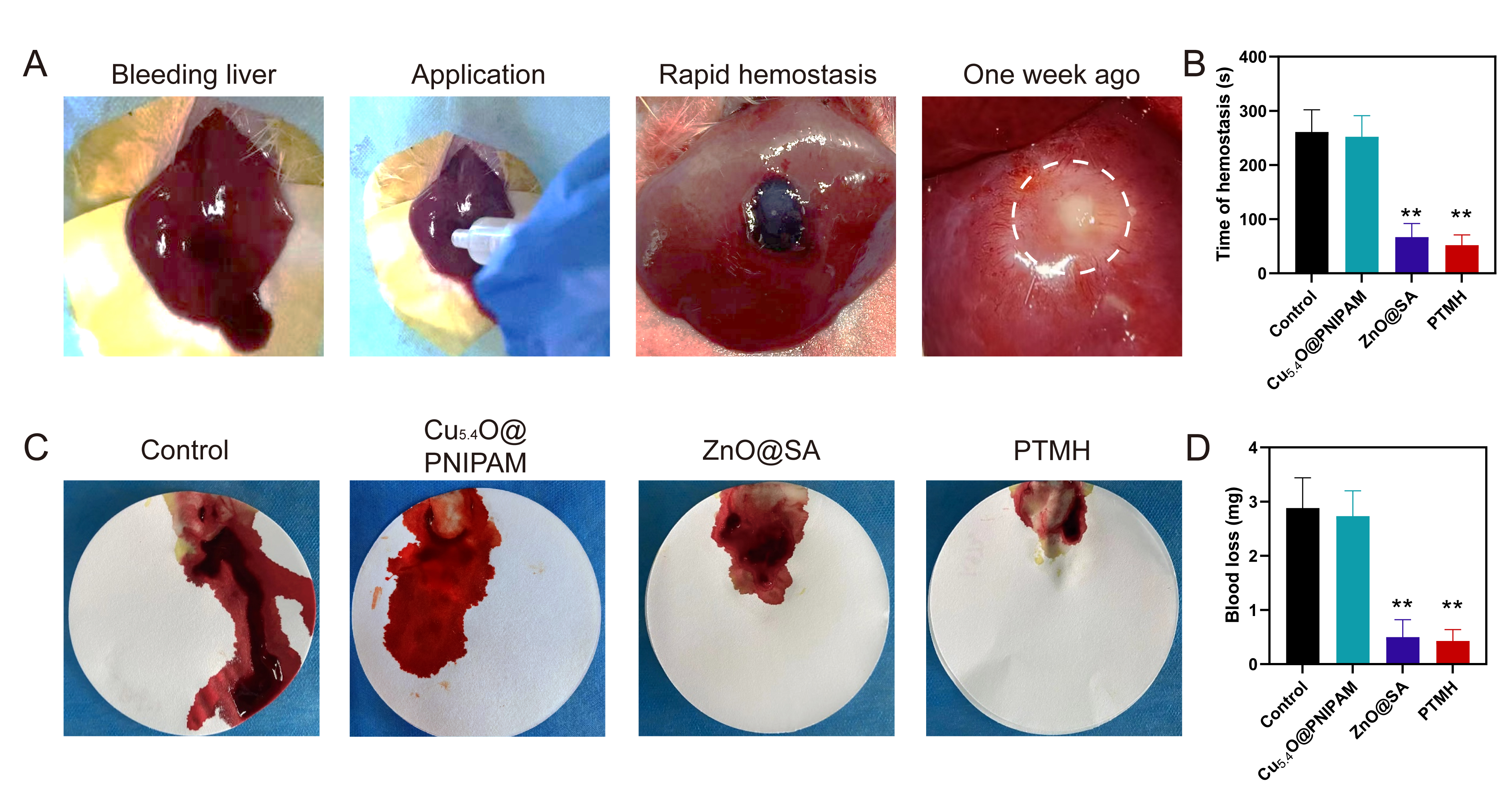


**Figure S8.** (A) Representative images of hemostatic assay using the hemorrhaging liver mouse model. (B) The time of hemostasis of different groups. (C) Representative images and (D) statistics analysis of blood loss. Data in B and D represent the mean ± standard deviation from five independent replicates (n = 5). ***p* < 0.01, One-way ANOVA.


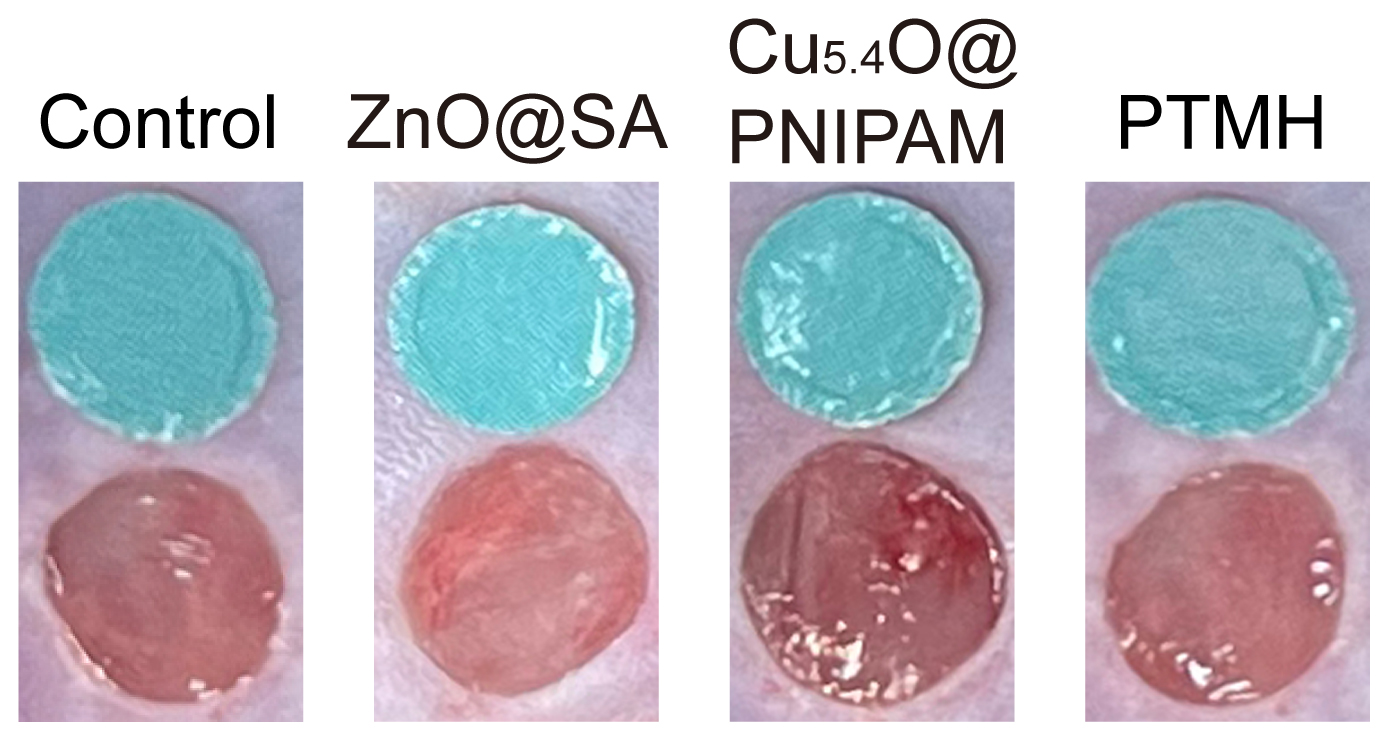


**Figure S9.** Representative images of diabetic wounds at day 0 post-treatment (blue 6-mm-diameter disc provided for scale reference).


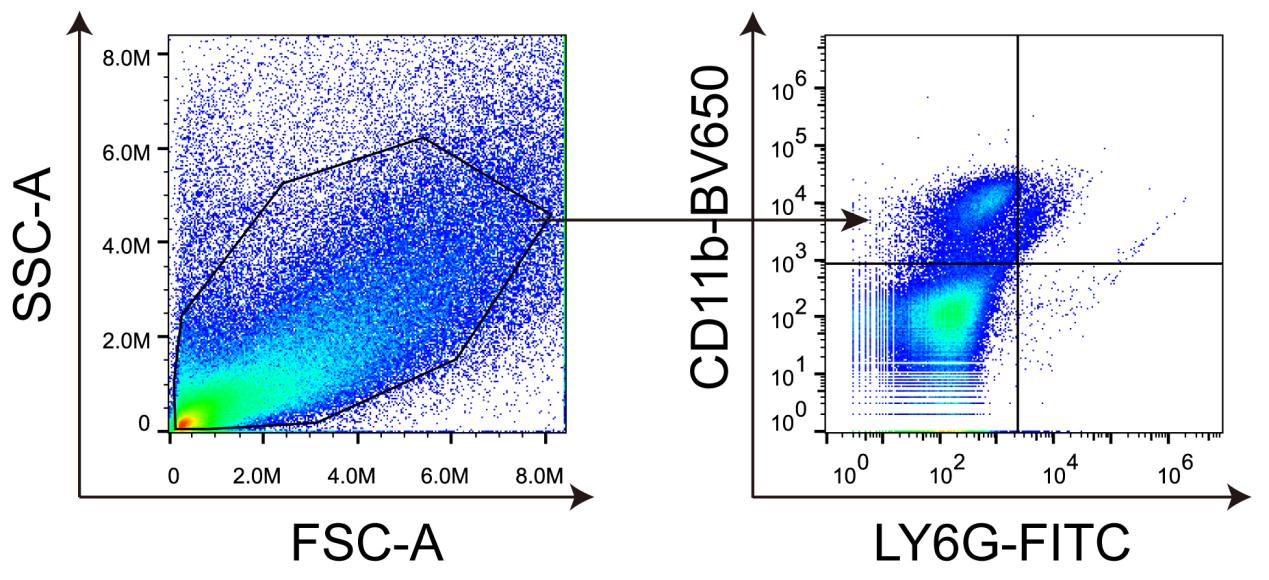


**Figure S10.** CD11b-Brilliant Violet 650^TM^(+)/LY6G-FITC(-) population were selected following FSC and SSC gating.


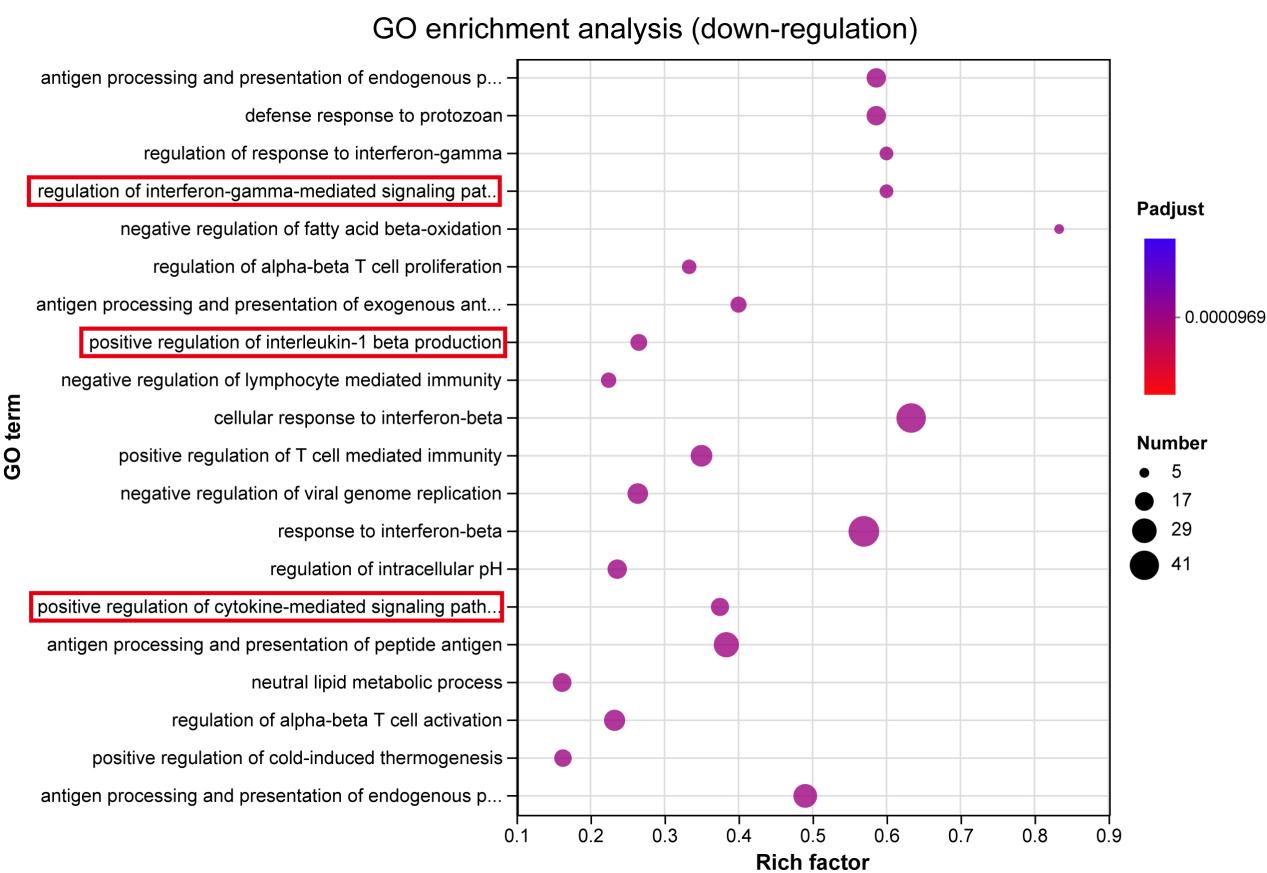


**Figure S11.** Gene ontology (GO) enrichment analysis of the differential genes (down-regulation) following PTMH treatment.


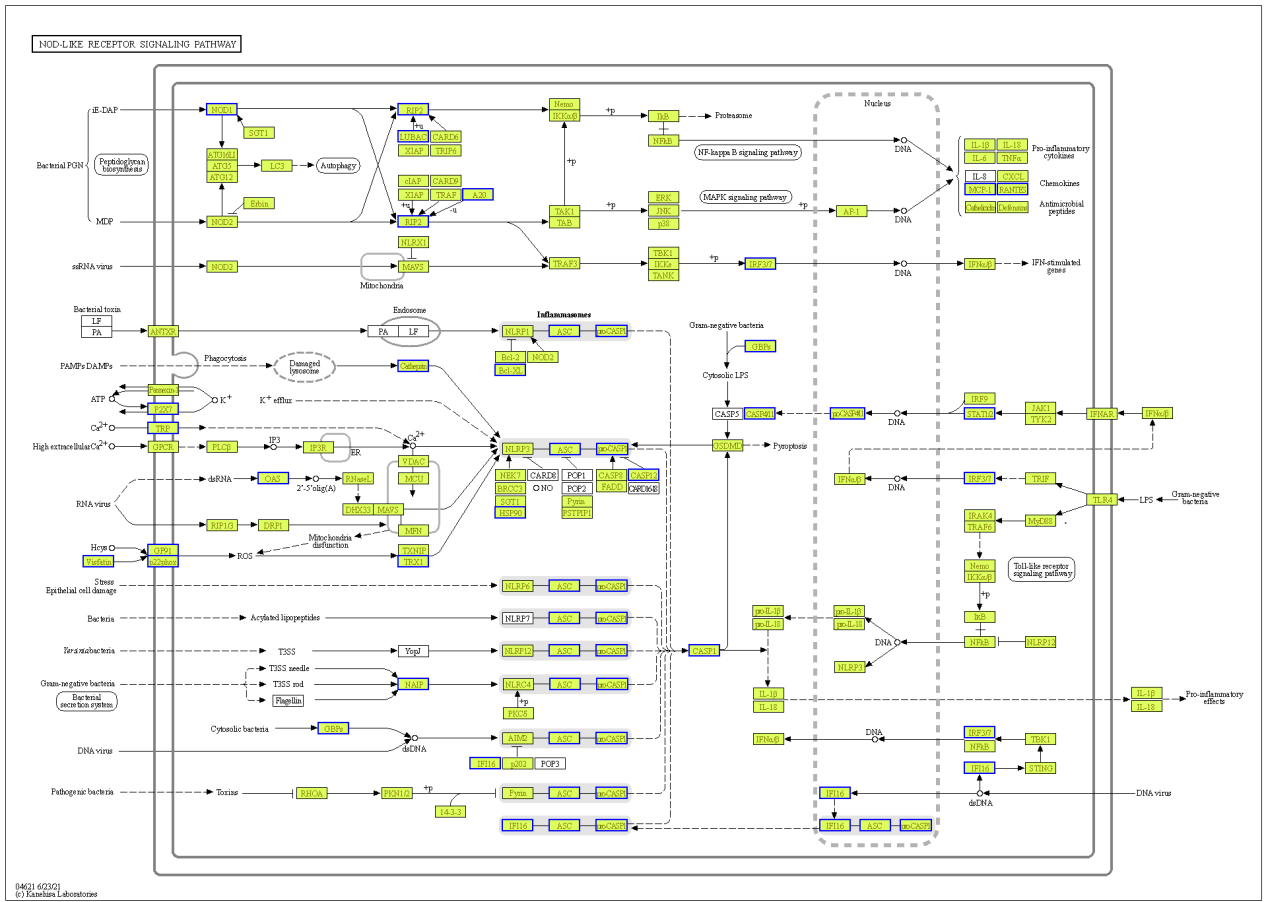


**Figure S12.** KEGG pathway enrichment analysis of differentially expressed genes (DEGs). NOD-like receptor signaling pathway. Blue outlines represented down-regulated DEGs.


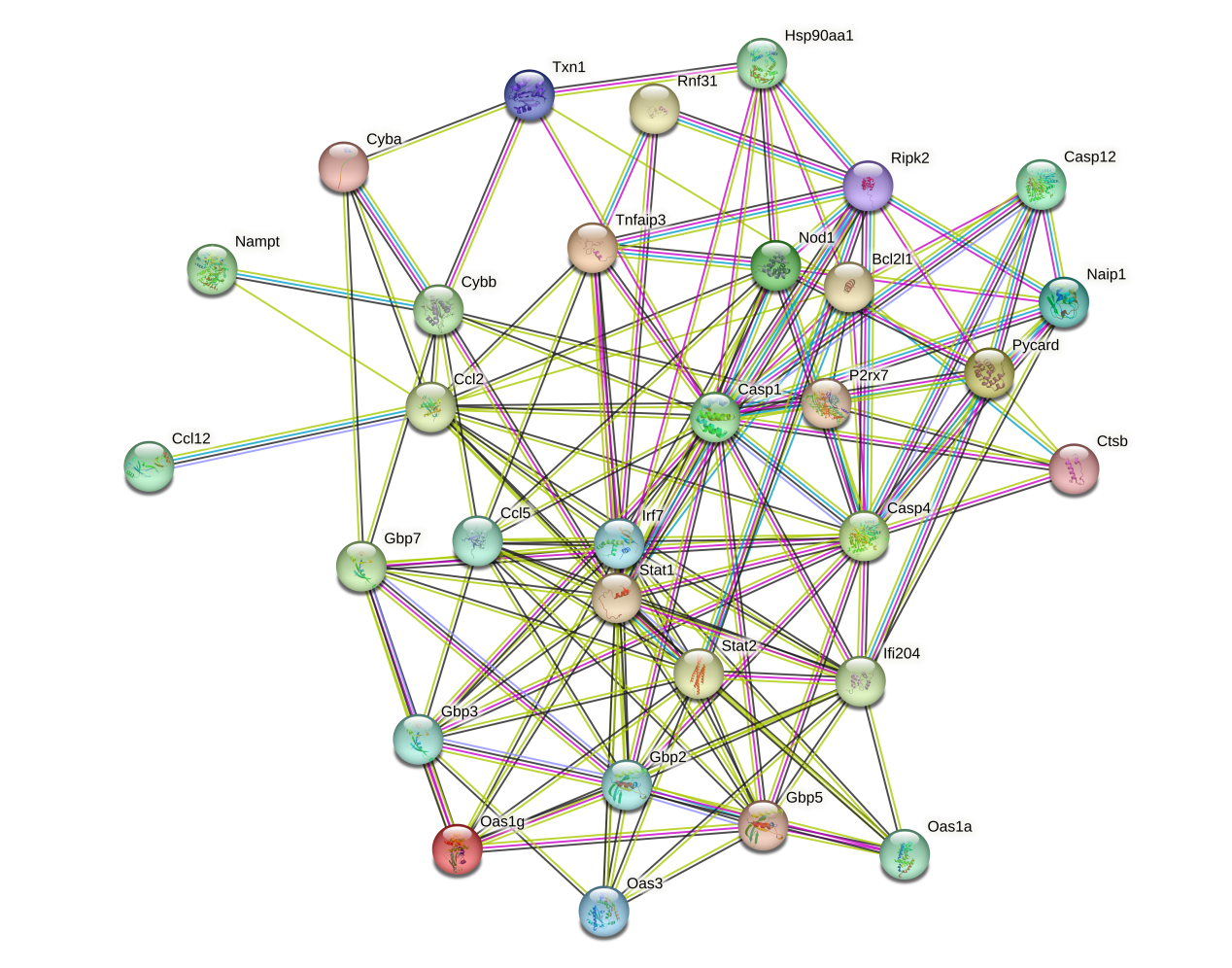


**Figure S13.** Protein-protein interaction (PPI) network of differential genes related with inflammation and oxidative stress that were significantly downregulated following PTMH treatment.


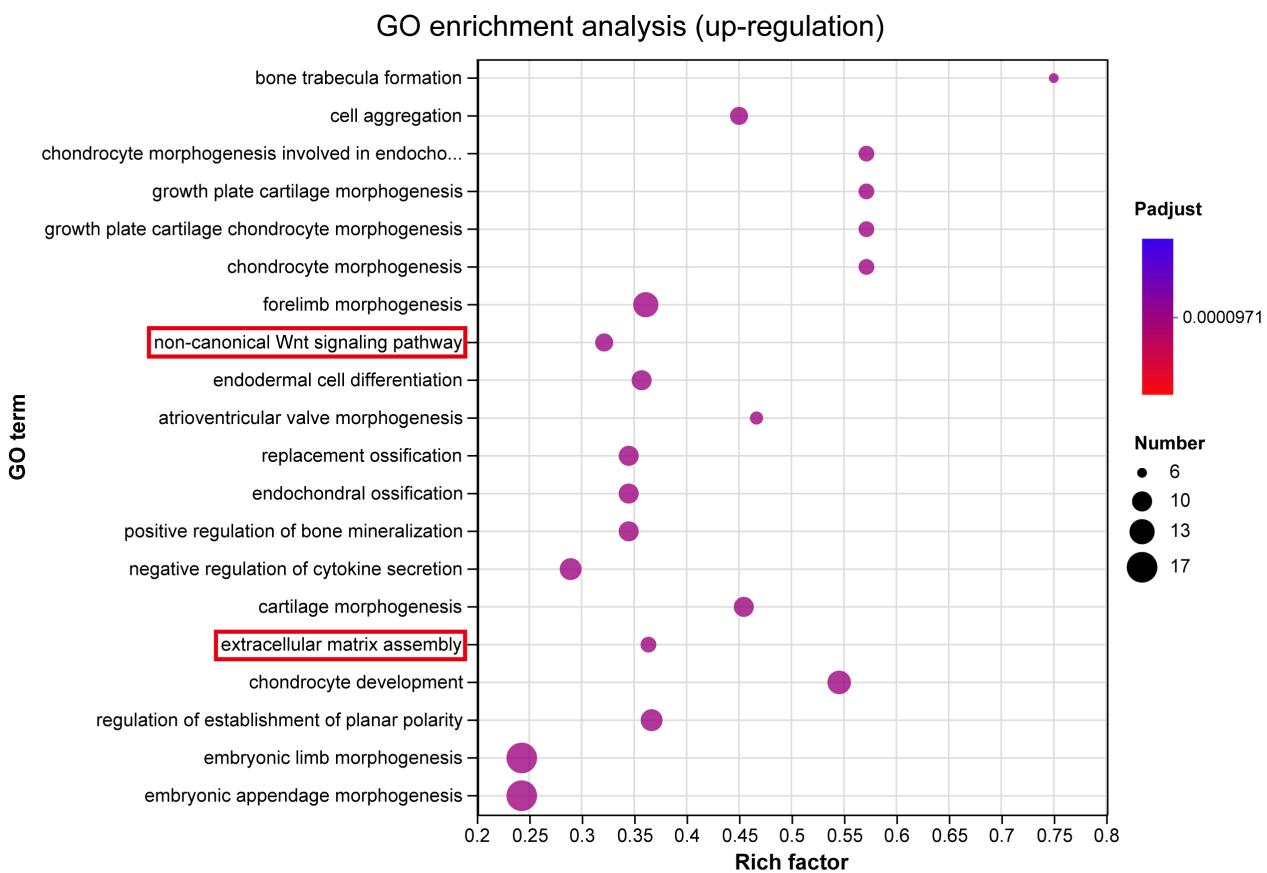


**Figure S14.** Gene ontology (GO) enrichment analysis of the differential genes (up-regulation) following PTMH treatment.


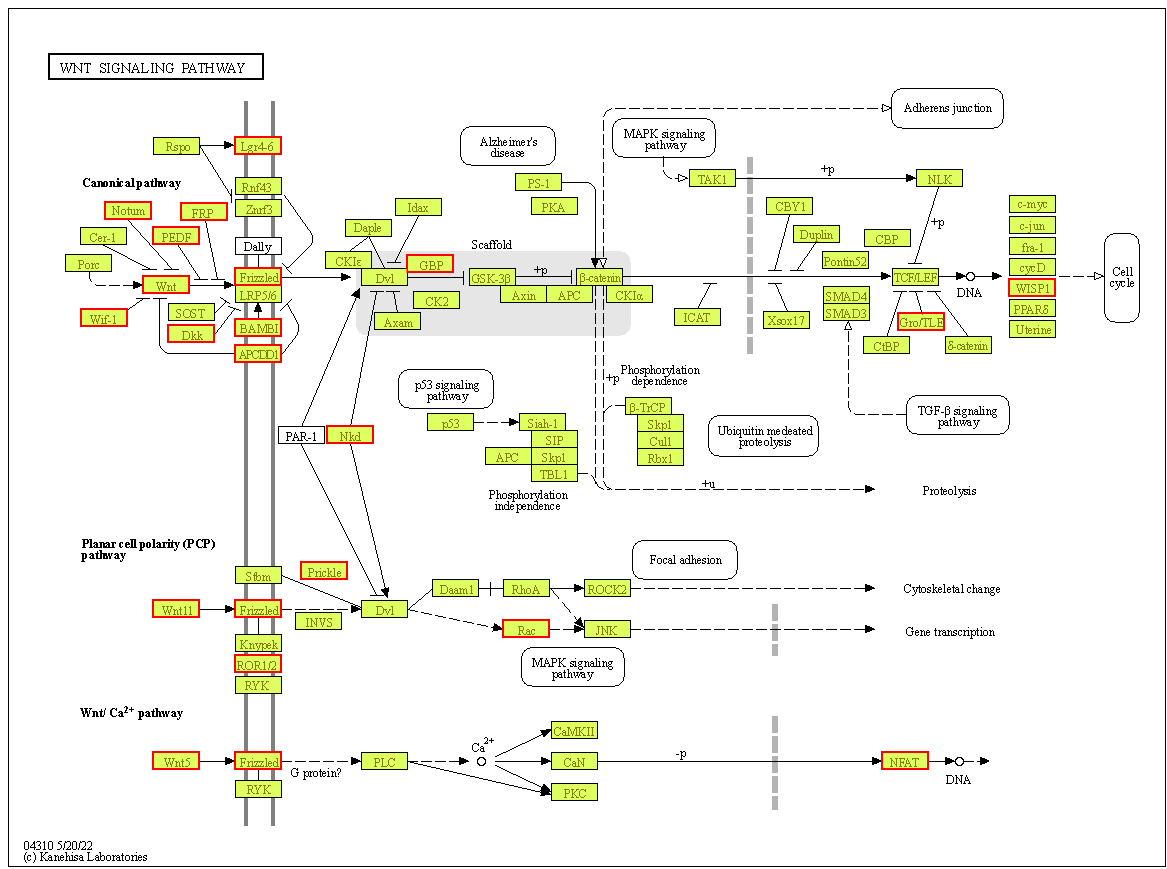


**Figure S15.** KEGG pathway enrichment analysis of differentially expressed genes (DEGs). Wnt signaling pathway. Red outlines represented upregulated DEGs.


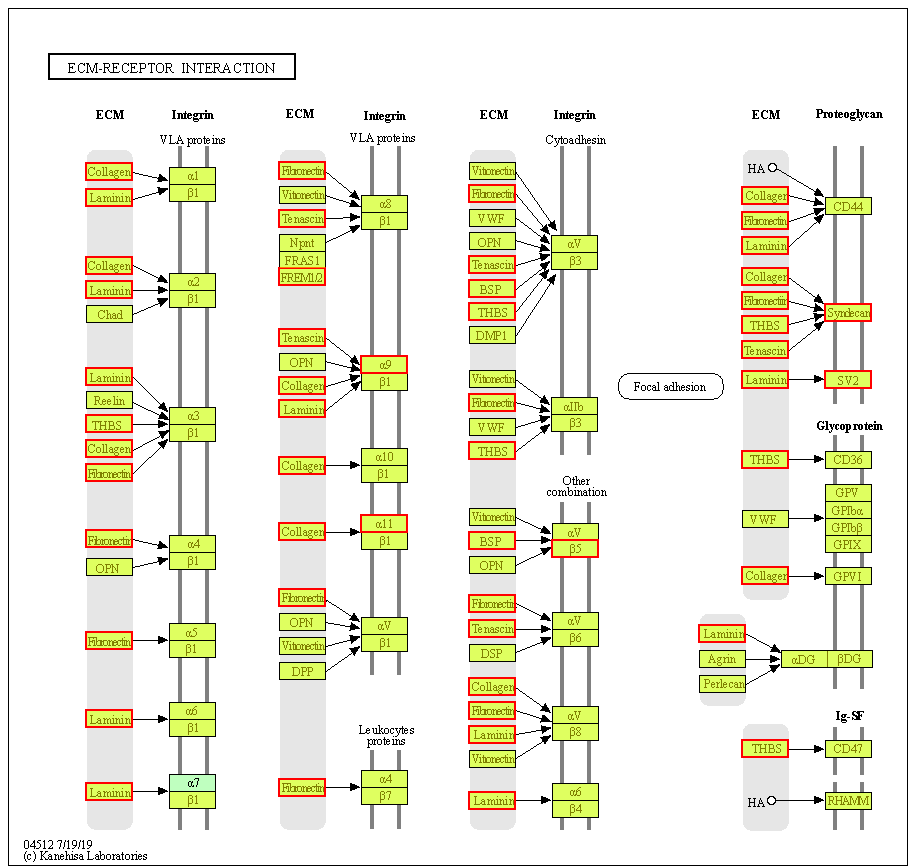


**Figure S16.** KEGG pathway enrichment analysis of differentially expressed genes (DEGs). Extracellular matrix (ECM)-receptor interaction. Red outlines represented upregulated DEGs.


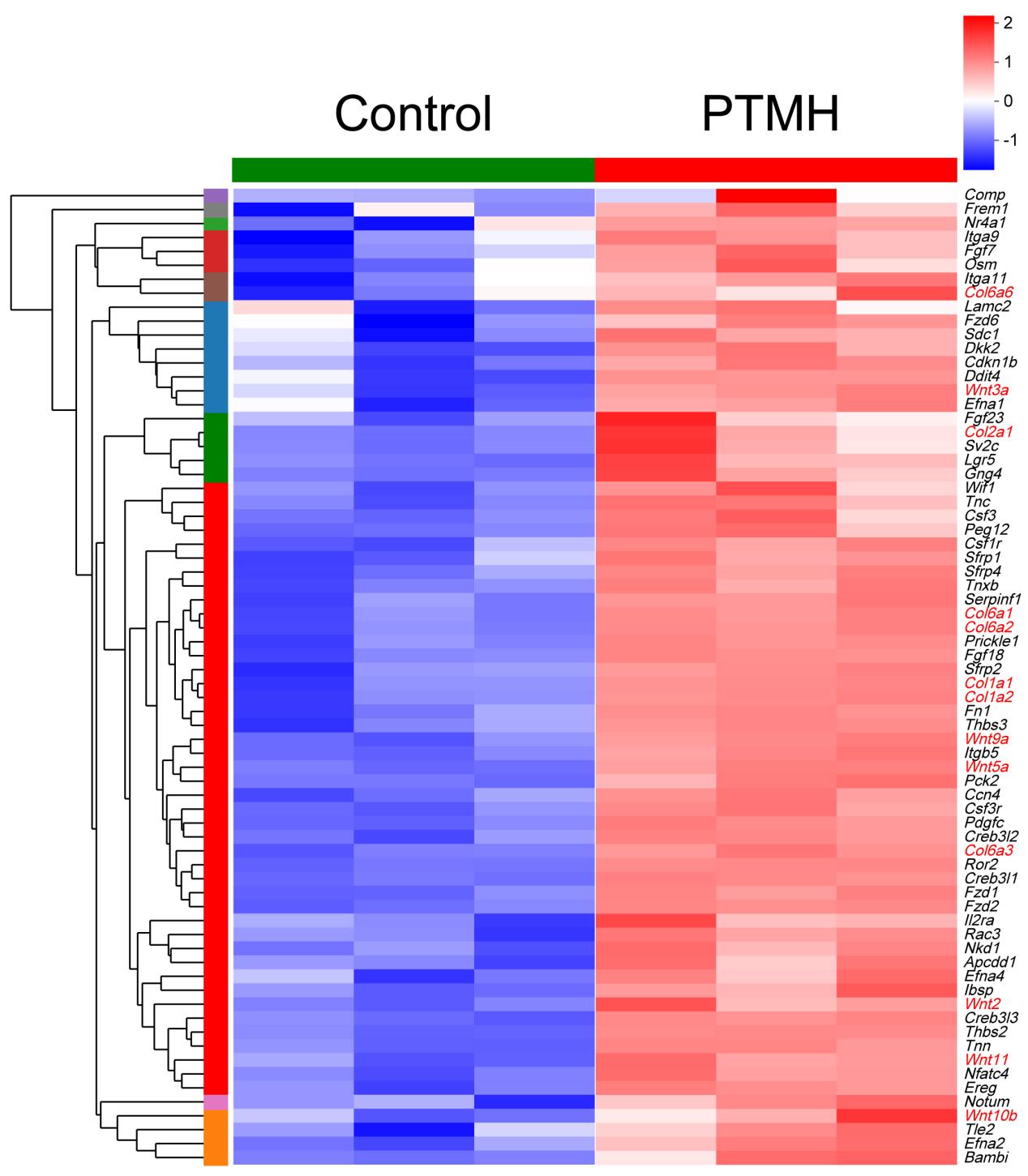


**Figure S17.** Heatmaps showing the genes involved in wound healing that were significantly upregulated following PTMH treatment.


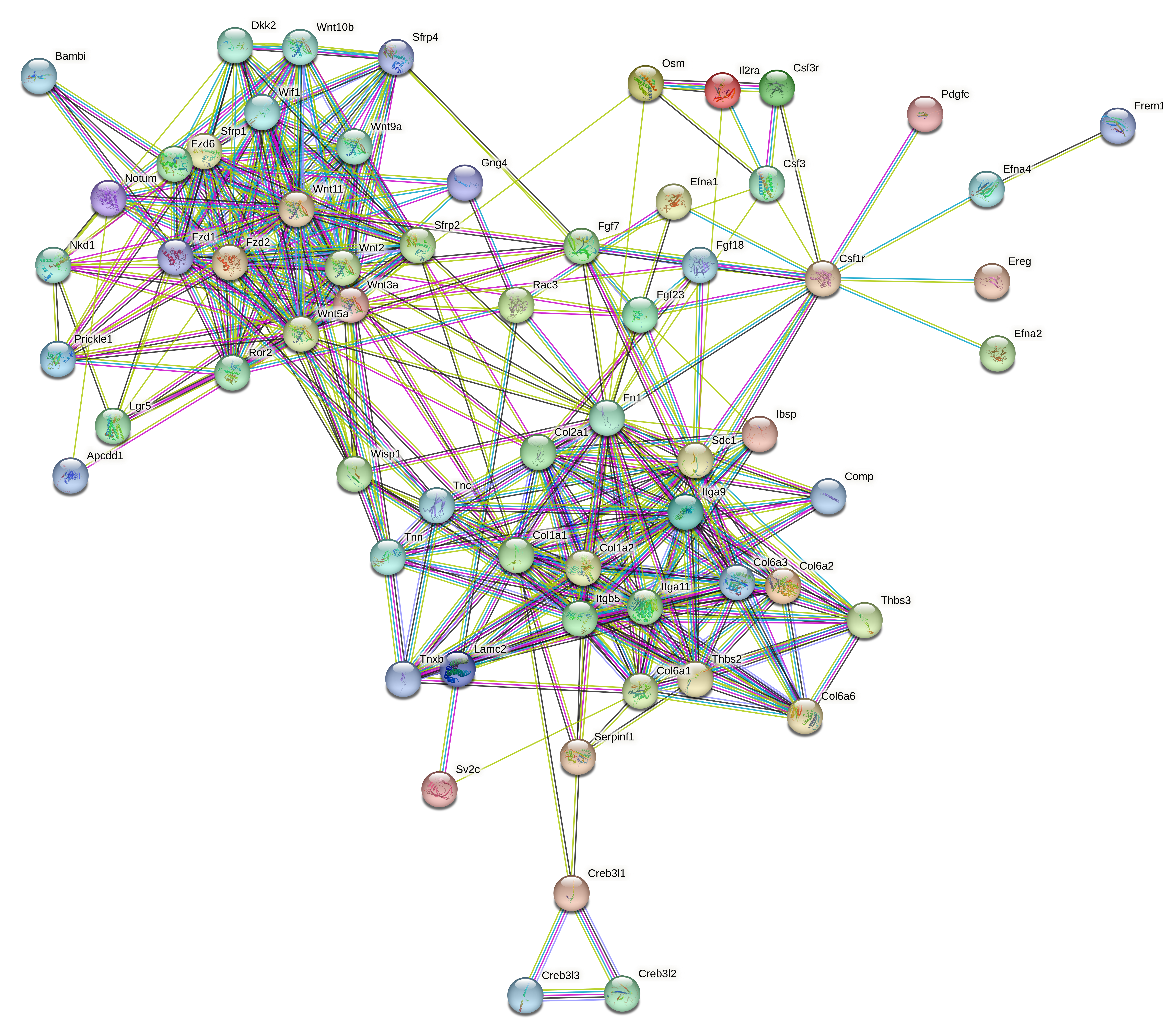


**Figure S18.** Protein-protein interaction (PPI) network of differential genes related with wound healing that were significantly upregulated following PTMH treatment.


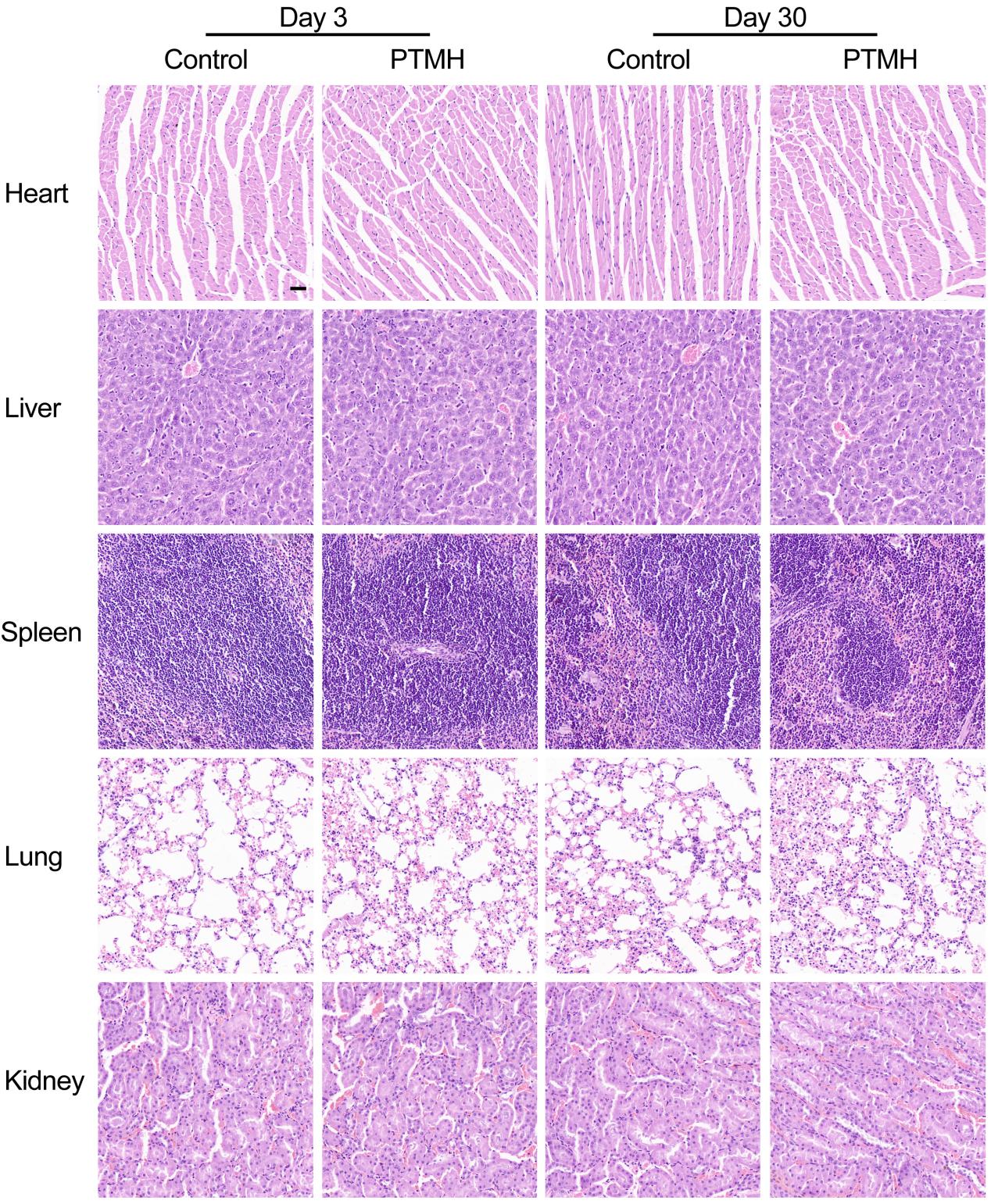


**Figure S19.** *In vivo* biocompatibility assessment of PTMH by major organs H&E sections. Evaluations of *in vivo* toxicity of PTMH to major organs (heart, liver, spleen, lung and kidney) at 3 days and 30 days under different treatments. (Scale bar: 50 μm)


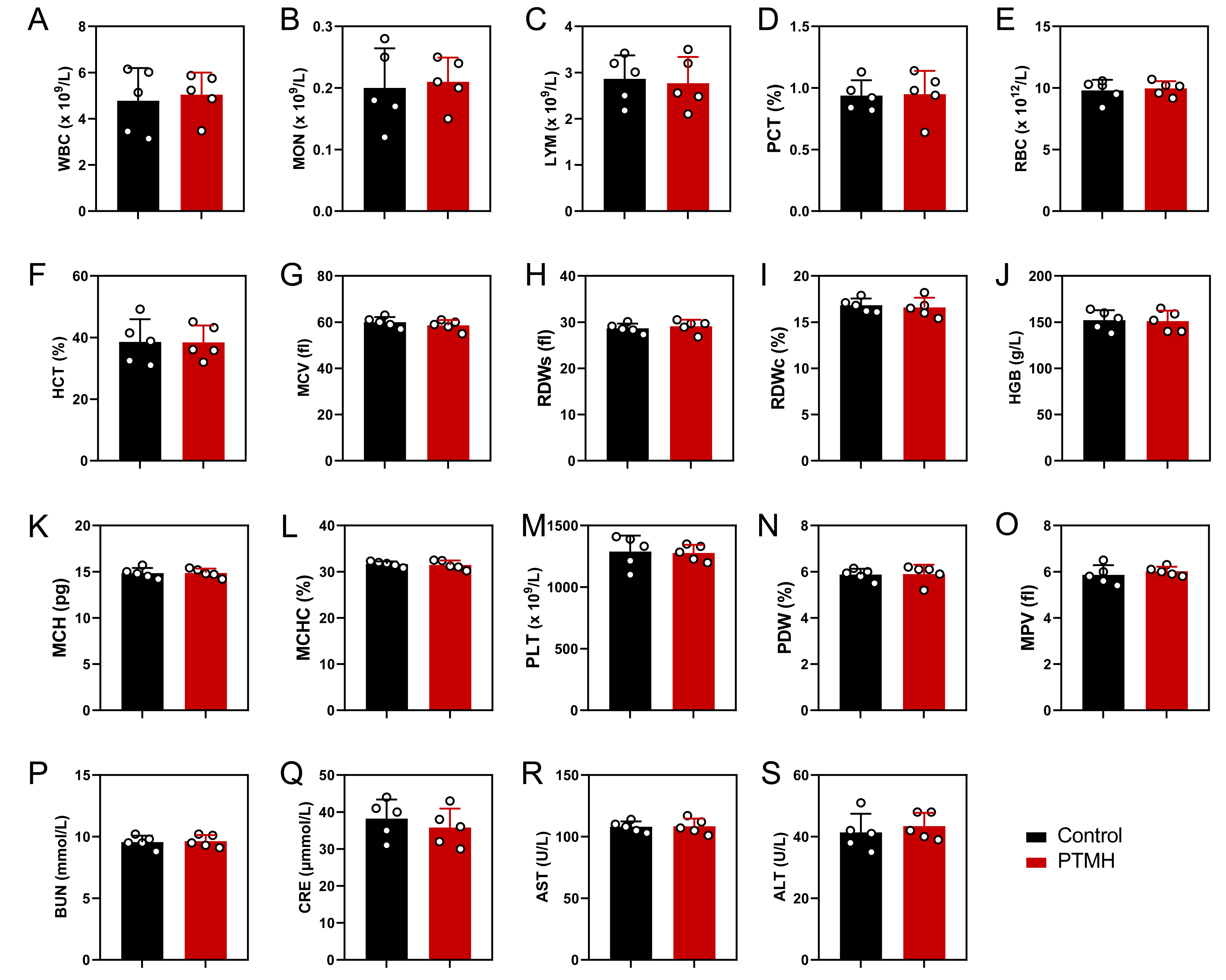


**Figure S20.** Biocompatibility assessment of PTMHs in *vivo*. (A) Serum levels of kidney function indicators: creatinine (CRE) and (B) blood urea nitrogen (BUN); (C) Serum levels of liver function indicators: alanine transaminase (ALT) and aspartate transaminase (AST); (D-H) Blood parameters in normal mice and with different treatments for 30 days. Data represent means ± s.d. from five independent replicates.
